# Spillover effects of RMB exchange rate among RCEP member countries: Empirical evidence from time-frequency domain approach

**DOI:** 10.1371/journal.pone.0287566

**Published:** 2023-06-23

**Authors:** Jingbo Guo, Zhiyong Wang

**Affiliations:** 1 School of Economics, Yunnan University of Finance and Economics, Kunming, Yunnan Province, China; 2 School of Finance, Yunnan University of Finance and Economics, Kunming, Yunnan Province, China; Universiti Malaysia Sabah, MALAYSIA

## Abstract

This study employs time-frequency domain approach to investigate the spillover effects of renminbi (RMB) exchange rate among the Regional Comprehensive Economic Partnership (RCEP) member countries. Utilizing daily data spanning from August 2010 to August 2022, we find that currencies in the RCEP region demonstrate significant interaction, which is primarily driven by short-term spillover, and ascend in response to major economic and political events. With respect to the influence of RMB, it displays frequency bands heterogeneity. Specifically, RMB tends to be a net receiver in the short term, but it primarily functions as a net transmitter in the long term. Notably, our analysis of time-varying spillover effects indicates that both domestic exchange rate regime reforms and external political and economic shocks amplify the net spillover effects of the RMB, which may be reflected in short-term connectedness or captured by long-term connectedness.

## 1. Introduction

On 15 November 2020, the Regional Comprehensive Economic Partnership (RCEP) was officially signed by 15 member countries, including ASEAN 10, China, Japan, South Korea, Australia, and New Zealand. As the largest trading bloc in the world, RCEP member countries collectively represent approximately 30% of the global population, GDP, and trade volume [1]. It is projected that the tariff concessions and investment liberalization in RCEP will lead to a $428 billion increase in intra-regional trade [2]. In light of complex international circumstances such as the COVID-19 pandemic, sluggish economic recovery, China-US trade tensions, and geopolitical conflicts, RCEP holds great significance in promoting comprehensive and sustainable development, as well as economic integration in the Asia-Pacific region [3].

In global and regional development, economic integration often goes hand in hand with financial integration [4]. The foreign exchange market is the largest financial market in the world with an enormous trading volume and uninterrupted operation, which makes it more sensitive to political and economic changes [5]. This implies a greater possibility for cross-border transmission of exchange rate risk. However, in the context of the RCEP region, only a limited amount of recent literature pays attention to the stock and energy markets [1, 6], with few studies focusing on the interlinkages of exchange rates among member countries, let alone the role of the RMB.

The transmission of exchange rate fluctuations stems from broad economic and financial linkages, primarily through three key channels: trade, finance, and sentiment [5, 7–12]. Firstly, trade integration fosters the emergence of regional currency blocs, and the competitiveness of trade further facilitates the co-movement of currencies [8, 13]. Secondly, globally active financial interlinkages can amplify cross-border capital flows, encompassing foreign direct investment and other types of financial transactions, thereby intensifying exchange rate fluctuations and cross-border contagion of risks. Thirdly, influenced by bounded rationality, investors’ trading behavior is susceptible to emotional influences. Empirical studies provide substantial evidence that investor sentiment possesses the capacity to impact and forecast exchange rates [12, 14–16]. Considering the extent of globalization in financial markets, sentiment effects are expected to spill over to multiple markets through cross-border capital flows or the dissemination of information across markets [12, 17]. Furthermore, it is crucial to comprehend the frequency dynamics of exchange rate linkages, as the shocks emanating from different channels exert varying frequencies and intensities of impact on connectedness [18, 19]. For instance, the trade channel typically exerts its influence over the long term, while the confidence effects and psychological expectations of investors transmit more rapidly. On the one hand, China is the largest economy and contributor to trade in the RCEP region, RCEP economies have a greater incentive to closely monitor the fluctuations of renminbi (RMB) in order to mitigate exchange rate risk [20, 21]. On the other hand, China’s capital account has not yet been fully opened and RMB has not become a widely-used funding currency internationally [9], which may limit the spillover effects of RMB. More importantly, the RCEP region not only possesses immense growth potential but also serves as a region where the internationalization of RMB is progressing at a rapid pace [2, 21]. This is evident in the outstanding progress made in areas such as cross-border RMB settlement, the development of offshore markets, and bilateral swap lines. Therefore, it is extremely essential to study the spillover effects of the RMB among RCEP member countries, particularly the differences between long-term and short-term dynamics.

The literature on exchange rate markets connectedness is quite extensive, encompassing the interlinkages among currencies of developed countries [22–26], spillover effects of developed country currencies to emerging market currencies [5, 27, 28], and the interdependence between currencies and other categories of assets, as stocks [29, 30], oil [31, 32], other commodities [31, 33, 34], and cryptocurrencies [35]. As China’s influence in the International Monetary System (IMS) continues to grow, there is an increasing body of literature that examines the interlinkages between the RMB and other currencies.

Our research closely aligns with the following two strands of literature. The first is based on the external currency model proposed by Frankel and Wei in 1994 [36], which estimates the implicit weight of the RMB in a basket of pegged currencies. Due to econometric issues such as multicollinearity and omitted variables in the benchmark model, empirical evidence on the influence of RMB is mixed [37–39]. While some researchers argue that RMB already has significant influence [7, 11, 40], others doubt the existence of RMB bloc [13, 41–44]. Also, the external currency model only considers the impact of RMB on other currencies and ignore the impact of other currencies on RMB. Another strand of the literature is based on the Vector Autoregression (VAR) model, using the spillover index approach proposed by Diebold and Yilmaz [45, 46] to measure the spillover effect between RMB and other currencies. According to Wei et al. [8] and Zhou et al. [47], the spillover effects of RMB have increased since the introduction of the Belt and Road Initiative. Furthermore, the influence of RMB is closely related to trade, financial linkages, and exchange rate regimes [5, 48]. The spillover index approach proposed by Diebold and Yilmaz [45, 46] can measure the directional spillovers and to some extent compensate for the shortcomings of the external monetary model, which is one of the motivations for choosing this methodology. Indeed, the approach of Diebold and Yilmaz [45, 46] has been widely applied in various research domains within finance and economics, unveiling the spillover effects among markets. However, the mean-based vector autoregression (VAR) approach proposed by Diebold and Yilmaz [45, 46], while widely used, fails to differentiate between positive and negative spillover effects. It also overlooks the varying information content of different frequency data and does not effectively capture market connectedness in extreme scenarios. Some literature has proposed asymmetric spillovers [49–51], frequency-domain spillovers [18, 52–54], tail dependence [55–58], and joint spillover index [58–60] to refine and extend the original spillover approach. Currently, most research on RMB spillover effects only considers time-domain spillovers. As previously mentioned, the causes of exchange rate markets connectedness are diverse and complex, with some acting in the short term and others taking longer. Therefore, it is essential to investigate short- and long-term spillover effects of RMB using the frequency-domain connectedness approach [18]. This is another motivation for adopting the spillover index approach. It is worth noting that the existing literature on the dynamic RMB spillover index mainly adopts the rolling window approach, which is sensitive to window selection and causes the loss of observations [61, 62].

We utilized time-frequency domain approach to investigate the spillover effects of RMB exchange rate among RCEP member countries. This study provides following potential marginal contributions: (i) enriching the research on the regional impact of RMB. Previous studies on RMB spillover effects have mainly focused on ASEAN and Belt and Road countries. To our knowledge, there has been no research on the spillover effects of RMB in the RCEP region. RCEP holds significant importance in promoting economic integration in the Asia-Pacific region during the COVID-19 pandemic [3], and the weakened influence of the US dollar in the RCEP region provides an opportunity for RMB to become an anchor currency [21]. Further exploration of the correlation between RMB and these currencies is crucial. (ii) We decompose the spillover effects into short-term and long-term, which compensates for the bias of solely studying in the time domain and facilitates the exploration of the heterogeneity of RMB influence in the long-term and short-term channels. (iii) We conducted a comparative analysis of the spillover effects of onshore RMB (CNY) and offshore RMB (CNH). Although the offshore RMB market is relatively free and prosperous in financial products [11], there has been relatively little research on the CNH spillover effects. The BIS Quarterly Review observed a surge in offshore RMB transactions between April 2019 and April 2022 [63], highlighting the importance of including CNH in the analysis. Additionally, we utilized time-varying parameters (TVP) to identify the dynamic evolution of spillover effects, which compensates for the limitations of rolling windows.

The findings of the study demonstrate that there exist significant interrelationships between currencies in the RCEP region, primarily driven by short-term spillover effects that increase during significant economic and political events. Regarding the spillover effects of the RMB, it primarily plays the role of a net receiver in the short term, while mainly acting as a net transmitter in the long term. This suggests that the influence of RMB is primarily achieved through long-term channels such as international trade and foreign direct investment. From a time-varying perspective, both domestic exchange rate regime reforms and external political and economic shocks increase the net spillover effect of the RMB, which may be reflected in short-term connectedness or captured by long-term connectedness. Consistent with the research of Zhou et al. [47], the influence of the RMB has increased following the "8.11" exchange rate regime reform. The changes in the market itself triggered by the China-US trade war were reflected in the evolution of spillovers in the long term, while the onset of the COVID-19 pandemic caused a short-term shock mainly through the channels of confidence and expectation. Compared to CNY, CNH exhibits a greater net spillover effect. However, during times of significant economic and political shocks, the directional spillover effect of CNY to remaining currencies experiences a higher growth rate. Our research findings aid in properly assessing the dynamic evolution of exchange rate risks and the heterogeneity of frequency bands, allowing for better prevention of risk resonance caused by cross-border contagion, and promoting deeper cooperation and sustainable development within the RCEP region. Moreover, our findings provide recommendations for enhancing the international influence of the RMB.

The remainder of this paper is structured as follows: Section 2 presents the research methodology and statistical analysis of the data. In Section 3, the empirical results are discussed. Finally, Section 4 provides the conclusion and implications of this study.

## 2. Methodology and data

### 2.1 Methodology

In this paper, the dynamic spillover index method proposed by Chatziantoniou et al.[62] is carried out for the empirical analysis, which extends the studies of Antonakakis et al.[61] as well as those of Baruník and Křehlík [18]. It should be noted that Antonakakis et al.[61] incorporate the spillover index proposed by Diebold and Yilmaz [45, 46] and the Time-Varying Parameter Vector Autoregression (TVP-VAR) model of Koop and Korobilis [64] as part of their study, while Chatziantoniou et al.[62] further combine the frequency domain spillover index of Baruník and Křehlík [18] with the TVP-VAR model thereby accomplishing the goal of measuring various time varying spillover indices.

#### 2.1.1 time-varying spillover index in time domain

We begin by building a TVP-VAR model as follows:

$$x_{t}=\Phi_{1t}x_{t-1}+\Phi_{2t}x_{t-2}+\cdots +\Phi_{pt}x_{t-p}+\epsilon_{t}\;\epsilon_{t}∼N(0,\Sigma_{t})$$ (1)

where *x*_*t*_ and *ϵ*_*t*_ are the N-dimensional column vectors, representing the exchange rate variable and error vector, Σ_*t*_ stands for a time-varying variance-covariance matrix (*N*×*N*), and Φ_*it*_, *i* = 1,…,*p* is also time-varying, denoting an *N*×*N* coefficient matrix.

If the above TVP-VAR variance-covariance process is stationary, then it can be simplified into the following TVP-VMA (*∞*) process.

$$x_{t}=\Psi (L)\epsilon_{t}$$ (2)

where $\Psi (L)=[\Phi (L){]}^{-1},\Phi (L)=[I_{N}-\Phi_{1t}L-\ldots -\Phi_{pt}L^{p}]$, and *L*^*p*^ is identity matrix.

Following Diebold and Yilmaz [45, 46], we adopt the generalized forecast error variance decomposition (GFEVD) since it is not influenced by the ordering of the variables. According to the principle of GFEVD, the contribution of the *j*th variable to the variance of the forecast error of the *i*th variable at the H-step forecasting horizon can be expressed in the following form:

$$\theta_{ijt}(H)=\frac{{\left(\Sigma_{t}\right)}_{jj}^{-1}\sum_{h=0}^{H}\;{\left({\left(\Psi_{h}\Sigma_{t}\right)}_{ijt}\right)}^{2}}{\sum_{h=0}^{H}\;{\left(\Psi_{h}\Sigma_{t}\Psi_{h}^{\prime }\right)}_{ii}}$$ (3)

Since $\sum_{i=1}^{N}\;\theta_{ijt}(H)\neq 1$, we have to normalize Eq (3). The normalized output is given as:

$${\tilde{\theta }}_{ijt}(H)=\frac{\theta_{ijt}(H)}{\sum_{j=1}^{N}\;\theta_{ijt}(H)}$$ (4)

such as $\sum_{i=1}^{N}\;{\tilde{\theta }}_{ijt}(H)=1$ and $\sum_{j=1}^{N}\;\sum_{i=1}^{N}\;{\tilde{\theta }}_{ijt}(H)=N$. Hence, all variables explain 100% of the variance of the forecast error of the *i*th variable.

We can then compute all of the spillover indices, beginning with the total spillover index (TSI). Here is the expression:

$${TSI}_{t}(H)=100\frac{\sum_{i\neq j}\;{\tilde{\theta }}_{ijt}(H)}{\sum {\tilde{\theta }}_{ijt}(H)}=100\left(1-\frac{\mathrm{Tr}\left\{{\tilde{\theta }}_{ijt}(H)\right\}}{N}\right)$$ (5)

where *Tr*{⋅} is the trace function. With the aim of identifying the direction of spillover throughout the network, next, we introduce three total directional spillover indices:(i) The total directional spillover index to others (TO) measures the total spillover from variable *i* to all other variables:

$$TO_{it}(H)=100\sum_{i=1,i\neq j}^{N}\;{\tilde{\theta }}_{jit}(H)$$ (6)

(ii) The total directional spillover index from others (FROM) measures the total spillover from all other variables to variable *i*:

$$FROM_{it}(H)=100\sum_{j=1,i\neq j}^{N}\;{\tilde{\theta }}_{ijt}(H)$$ (7)

(iii) The net total directional spillover index (NET) equals the total directional spillover index to others (To) minus the total directional spillover index from others (From), i.e.,

$${\mathrm{NET}}_{\mathrm{it}}\left(H\right)=TO_{it}(H)-FROM_{it}(H)$$ (8)

if *NET*_*it*_(*H*)>0, variable *i* is a net transmitter in the whole system and vice versa.

In addition, we sometimes need to measure the connectedness between two variables, which can be defined as

$$C_{ijt}(H)={\tilde{\theta }}_{ijt}(H)-{\tilde{\theta }}_{jit}(H)$$ (9)

if *C*_*ijt*_(*H*)>0, variable *j* has more influence over variable *i* than variable *i* has over variable *j*, and vice versa.

#### 2.1.2 time-varying spillover index in frequency domain

To further explore the spillover effect at different frequency bands, we first define the frequency response function, $\Psi (e^{-i\omega })=\sum_{h=0}^{\infty }\;e^{-i\omega h}\Psi_{h}$, which represents the coefficients of the Fourier transform, with $i=\sqrt{-1}$. On this basis, the spectral density of *x*_*t*_ at frequency ω can be expressed as:

$$S_{x}(\omega )=\sum_{h=-\infty }\;E(x_{t}x_{t-h}^{\prime })e^{-i\omega h}=\Psi (e^{-i\omega h})\Sigma_{t}\Psi^{\prime }(e^{+i\omega h})$$ (10)

As in the time domain, we prefer frequency GFEVD as follows,

$$\theta_{ijt}(\omega )=\frac{{\left(\Sigma_{t}\right)}_{jj}^{-1}{\left|\sum_{h=0}^{\infty }\;{\left(\Psi (e^{-i\omega h})\Sigma_{t}\right)}_{ijt}\right|}^{2}}{\sum_{h=0}^{\infty }\;{\left(\Psi (e^{-i\omega h})\Sigma_{t}\Psi (e^{i\omega h})\right)}_{ii}}$$ (11)

Where *θ*_*ijt*_(*ω*) denotes the contribution of *j*th variable to the portion of the spectrum of *i*th variable at the frequency *ω*. After normalizing it, the expression is as follows:

$${\tilde{\theta }}_{ijt}(\omega )=\frac{\theta_{ijt}(\omega )}{\sum_{k=1}^{N}\;\theta_{ijt}(\omega )}$$ (12)

Typically, we are interested in spillover effects within a particular band of frequencies, rather than at any particular frequency. Thus, given a random frequency band: *d* = (*a*, *b*): *a*, *b*∈(−*π*, *π*), *a*<*b*, the frequency GFEVD at frequency band *d* can then be expressed as

$${\tilde{\theta }}_{ijt}(d)=\int_{a}^{b}\Gamma_{i}(\omega )\;{\tilde{\theta }}_{ijt}(\omega )d\omega$$ (13)

where *Γ*_*i*_(*ω*) stands for the weighting function. Based on the above study, we can calculate accurately the various spillover indices at the frequency band *d*:

$${TSI}_{t}(d)=100\frac{\sum_{i\neq j}\;{\tilde{\theta }}_{ijt}(d)}{N}$$ (14)

$$TO_{it}(d)=100\sum_{i=1,i\neq j}^{N}\;{\tilde{\theta }}_{jit}(d)$$ (15)

$$FROM_{it}(d)=100\sum_{j=1,i\neq j}^{N}\;{\tilde{\theta }}_{ijt}(d)$$ (16)

$${\mathrm{NET}}_{\mathrm{it}}\left(d\right)=TO_{it}(d)-FROM_{it}(d)$$ (17)

$$C_{ijt}(d)={\tilde{\theta }}_{ijt}(d)-{\tilde{\theta }}_{jit}(d)$$ (18)

### 2.2 Data description and analysis

This article aims to analyze the time-frequency spillover effects of RMB exchange rate among RCEP countries. With the exception of currencies pegged to either the US dollar or Singapore dollar(i.e., Cambodian Riel and Brunei dollar) as well as those with a large amount of missing data (i.e., Myanmar Kyat and Laotian Kip), we employ a daily exchange rate dataset (against the US dollar) consisting of RMB and currencies of other ten RCEP members, namely the onshore RMB (CNY), the offshore RMB (CNH), the Japanese yen (JPY), the South Korea won (KRW), the Australian dollar (AUD), the New Zealand dollar (NZD), the Thailand baht (THB), the Singapore dollar (SGD), the Indonesian rupiah (IDR), the Philippines peso (PHP), the Malaysian ringgit (MYR) and the Vietnam Dong (VND). The sample period runs from 23 August 2010 to 19 August 2022, which is comprehensive and can present the RCEP development pathway since it was proposed in 2012. The starting point of the sample period is determined by the availability of CNH. All the data series are obtained from Bloomberg.

We compute the returns using the logarithmic difference on the raw exchange rate data, with negative (positive) returns reflecting the appreciation (depreciation) of the home currency. Table 1 presents the descriptive statistics results of the log returns for all currencies. Based on the direct quotation method, all the return series have positive means, implying that all currencies depreciate in the sample period. Most notably, CNY and CNH have a relatively small standard deviation, suggesting that onshore and offshore RMB are more stable than the other currencies (except for VND). Moreover, the Jarque–Bera test results show that all variables are non-normality at the 1% significance level. To be more explicit, the kurtosis of each market is greater than 3, showing a leptokurtic distribution. ADF tests indicate that all variables do not have unit roots (i.e., series are stationary), satisfying the conditions for conducting econometric analysis.

**Table 1:** Descriptive statistics results.

Currency	Mean	Std	Min	Max	Skewness	Kurtosis	Jarque-Bera	Q(5)	Q(20)	ADF
**CNY**	0.00016	0.229	-1.195	2.331	1.101	15.714	16822.940***	12.420**	39.267***	-49.435***
**CNH**	0.00068	0.271	-1.471	2.747	0.638	13.589	11916.520***	3.520	21.363	-50.078***
**JPY**	0.01896	0.609	-3.782	4.508	0.111	8.467	3135.529***	3.008	18.270	-50.279***
**KRW**	0.00457	0.548	-1.937	4.751	0.467	6.714	1536.633***	8.106	25.479	-50.115***
**AUD**	0.01040	0.722	-3.392	6.309	0.436	7.240	1963.154***	3.745	10.492	-50.274***
**NZD**	0.00537	0.752	-3.113	5.684	0.366	5.779	865.197***	3.319	14.006	-51.017***
**MYR**	0.01422	0.431	-3.470	2.557	-0.266	8.830	3590.345***	22.330***	59.444***	-32.319***
**SGD**	0.00100	0.360	-2.381	2.670	0.376	8.360	3068.727***	3.848	20.144	-51.605***
**PHP**	0.00862	0.333	-2.530	1.940	0.070	6.701	1436.824***	17.035***	33.092***	-52.878***
**VND**	0.00729	0.174	-0.927	6.505	21.081	771.673	62078521***	3.342	8.160	-48.613***
**THB**	0.00499	0.348	-2.189	2.823	0.435	8.557	3313.482***	24.523***	42.809***	-46.455***
**IDR**	0.02002	0.455	-3.826	6.356	0.827	32.882	93819.530***	57.099***	94.474***	-43.625***

Note: ***, ** and * represent significance levels of 1%, 5% and 10%, respectively. ADF refers to unit-root test. J-B refers to the statistic of the Jarque-Bera test. Q(5) and Q(20) are the Ljung-Box Q statistics with lags of 5th and 20th order, respectively.

## 3. Empirical results and discussion

In this section, we estimate the spillover effects in both the time and frequency domains. The optimal lag length (*p*) for the generalized vector autoregression model is selected by the Akaike information criterion (AIC) and the Schwartz criterion (SC). Furthermore, in this paper, we decompose the spillover indices into two different frequency bands, namely d1 = (0,0.628), d2 = (0.628,3.142). We define them as high frequency (1–5 days) and low-frequency (more than 5 days), representing the short-term and long-term spillover effects respectively. According to the study of Barunik and Krehik [18], the length of the forecasting horizon (*H*) requires sufficiently large, otherwise the methodology will not work. Thus, referring to some recent studies [19, 65], we set the forecasting horizon (*H*) to 100 days.

### 3.1 Static spillover effects

#### 3.1.1 Static spillover effects between CNY and currencies of RCEP members

Tables 2 and 3 show the static spillover effects between CNY and currencies of RCEP members in the time and frequency domain, respectively. It is worth mentioning that elements on the main diagonal of Table 2 correspond to shocks on their own, while off-diagonal elements refer to interrelationships between currencies.

**Table 2:** Static spillover effect between CNY and currencies of RCEP members in time domain.

	CNY	JPY	KRW	AUD	NZD	MYR	SGD	PHP	VND	THB	IDR	FROM
**CNY**	55.58	1.00	5.97	6.16	5.11	4.88	8.63	1.89	0.26	6.40	4.12	44.42
**JPY**	1.41	80.75	0.54	3.43	5.28	0.06	7.52	0.06	0.01	0.71	0.22	19.25
**KRW**	4.22	0.74	36.71	9.24	7.51	9.83	13.30	5.78	0.21	7.28	5.18	63.29
**AUD**	3.65	1.47	4.75	34.37	22.92	4.77	19.64	1.56	0.04	4.01	2.81	65.63
**NZD**	3.25	2.39	3.85	24.27	36.47	3.92	18.43	1.20	0.08	3.75	2.39	63.53
**MYR**	3.52	0.48	9.66	8.61	6.63	36.76	13.46	5.63	0.32	7.55	7.37	63.24
**SGD**	4.72	3.00	6.52	18.19	16.15	7.89	31.89	2.55	0.15	5.77	3.17	68.11
**PHP**	1.89	0.45	8.10	5.02	4.10	8.32	7.66	53.98	0.16	6.16	4.16	46.02
**VND**	0.87	0.19	1.11	0.93	0.95	0.91	0.98	0.56	91.85	0.45	1.19	8.15
**THB**	4.78	1.48	7.57	7.81	6.94	8.48	10.77	4.55	0.15	41.29	6.17	58.71
**IDR**	3.83	0.47	6.91	6.89	5.03	9.90	7.45	3.63	0.62	7.40	47.87	52.13
**TO**	32.13	11.67	55.00	90.57	80.65	58.96	107.84	27.42	2.00	49.47	36.77	552.48
**NET**	-12.29	-7.58	-8.29	24.94	17.11	-4.28	39.73	-18.60	-6.15	-9.25	-15.35	**TSI = 50.23**

Note:(i) Results are based on the time-domain model with lag length (*p*) of 1 day and forecasting horizon (*H*) of 100 days. (ii) TSI is the total spillover index in the whole network.TO is the total directional spillover transmitting to the remaining currencies. FROM is the total directional spillovers received from all other currencies. NET is the difference between TO and FROM.

**Table 3:** Static spillover effect between CNY and RCEP currencies in frequency domain.

	CNY	JPY	KRW	AUD	NZD	MYR	SGD	PHP	VND	THB	IDR	FROM
**Panel A. Short-term spillover (1–5 days)**												
**CNY**	45.29	0.73	4.72	4.66	3.92	3.92	6.51	1.44	0.20	5.08	3.44	34.62
**JPY**	1.06	65.63	0.45	2.79	4.30	0.05	6.17	0.06	0.01	0.54	0.19	15.63
**KRW**	3.17	0.51	29.49	6.52	5.37	7.54	9.39	4.44	0.19	5.39	4.23	46.74
**AUD**	3.03	1.24	3.78	27.89	18.86	3.81	15.98	1.25	0.03	3.21	2.30	53.48
**NZD**	2.61	1.97	3.10	19.66	29.85	3.20	14.98	1.01	0.06	2.97	1.96	51.53
**MYR**	2.54	0.37	7.23	5.85	4.60	28.70	9.25	4.31	0.22	5.79	5.63	45.81
**SGD**	3.86	2.33	5.36	14.94	13.44	6.60	26.33	2.15	0.14	4.76	2.64	56.21
**PHP**	1.43	0.35	6.57	3.61	3.01	6.51	5.52	44.78	0.12	4.76	3.27	35.15
**VND**	0.53	0.13	0.68	0.58	0.58	0.55	0.54	0.34	73.54	0.27	0.78	4.99
**THB**	3.69	1.01	5.82	5.30	4.75	6.52	7.43	3.67	0.12	32.25	4.77	43.07
**IDR**	2.67	0.30	4.98	4.33	3.25	7.06	4.80	2.74	0.40	5.32	35.79	35.85
**TO**	24.58	8.93	42.69	68.25	62.07	45.75	80.58	21.41	1.51	38.10	29.22	423.08
**NET**	-10.04	-6.69	-4.05	14.76	10.54	-0.06	24.37	-13.74	-3.48	-4.97	-6.63	**TSI = 38.46**
**Panel B. Long-term spillover (more than 5 days)**												
**CNY**	10.29	0.27	1.25	1.51	1.19	0.96	2.12	0.46	0.05	1.32	0.67	9.80
**JPY**	0.35	15.11	0.08	0.63	0.99	0.01	1.35	0.00	0.00	0.17	0.03	3.63
**KRW**	1.05	0.24	7.23	2.72	2.14	2.30	3.91	1.35	0.02	1.88	0.95	16.55
**AUD**	0.62	0.24	0.98	6.48	4.07	0.96	3.65	0.31	0.00	0.80	0.51	12.14
**NZD**	0.64	0.42	0.75	4.61	6.61	0.72	3.45	0.19	0.01	0.77	0.43	12.01
**MYR**	0.98	0.11	2.43	2.75	2.04	8.06	4.21	1.31	0.10	1.76	1.74	17.43
**SGD**	0.86	0.67	1.16	3.25	2.72	1.29	5.56	0.40	0.02	1.01	0.53	11.90
**PHP**	0.46	0.10	1.54	1.41	1.09	1.82	2.14	9.20	0.04	1.39	0.88	10.87
**VND**	0.34	0.05	0.43	0.35	0.38	0.36	0.44	0.22	18.31	0.18	0.41	3.16
**THB**	1.09	0.48	1.76	2.52	2.19	1.95	3.34	0.88	0.03	9.03	1.41	15.64
**IDR**	1.15	0.17	1.94	2.56	1.78	2.84	2.65	0.88	0.22	2.08	12.08	16.27
**TO**	7.55	2.74	12.31	22.32	18.58	13.21	27.26	6.01	0.49	11.36	7.56	129.39
**NET**	-2.25	-0.89	-4.23	10.18	6.57	-4.22	15.36	-4.86	-2.67	-4.28	-8.72	**TSI = 11.76**

Note:(i) Results are based on the frequency-domain model with lag length (*p*) of 1 day and forecasting horizon (*H*) of 100 days. (ii) TSI is the total spillover index in the whole network.TO is the total directional spillover transmitting to the remaining currencies. FROM is the total directional spillovers received from all other currencies. NET is the difference between TO and FROM.

Beginning with the total spillover effect, we observe that the index is 50.23%, decomposing into 38.46% attributed to the short term and 11.76% to the long term. This finding suggests that the interaction among the whole currency markets is strong. What is more, it appears that the total spillover effect is driven by the short-term connectedness, which is consistent with the study of Anwer et al.[19]. To put differently, information transmissions tend to be faster within a short period of time.

Looking at the net total directional spillovers, SGD is the largest net spillover transmitter in the whole currency system (39.73%), followed by AUD (24.94%) and NZD (17.11%). This is in line with the findings of Bouri et al.[66] and Anwer et al.[19]. As one of the key members of ASEAN, Singapore has close cooperation with Southeast Asian countries. Moreover, Singapore has become a global financial center due to its favorable geographical location. As such, SGD has strong spillovers to other currencies. Australia is a major global supplier of commodities, which makes fluctuations in AUD more easily contagious to other currencies. In contrast, all other currencies are net spillover receivers. With reference to frequency bands, most currencies are driven by the short term, while MYR and IDR by the long term.

We focus our attention on CNY. As shown in Table 2, CNY is a net spillover receiver in the whole network, yet it acts as a net information transmitter to JPY and VND. Concerning the frequency bands, over the long run, CNY functions as a net information transmitter not only to JPY and VND, but also to MYR and IDR, which indicates a more pronounced impact of CNY. In brief, CNY exerts a specific influence over the currencies of RCEP members, which is heterogeneous in terms of both countries and frequency bonds.

**3.1.2 Static spillover effects between CNH and currencies of RCEP members.** To arrive at a more comprehensive analysis of the connectedness between RMB and currencies of RCEP members, we quantify the spillover effects of CNH based on the time-frequency domain approach, with the results shown in Tables 4 and 5.

**Table 4:** Static spillover effect between CNH and currencies of RCEP members in time domain.

	CNH	JPY	KRW	AUD	NZD	MYR	SGD	PHP	VND	THB	IDR	FROM
**CNH**	53.62	0.97	4.29	9.33	7.60	3.80	12.32	1.38	0.22	4.31	2.16	46.38
**JPY**	1.43	79.71	0.48	3.47	5.54	0.12	8.01	0.06	0.03	0.91	0.24	20.29
**KRW**	4.59	0.80	36.75	9.41	7.51	9.64	13.39	5.98	0.12	6.89	4.93	63.25
**AUD**	5.97	1.50	4.51	34.33	22.63	4.15	19.13	1.47	0.02	3.79	2.51	65.67
**NZD**	5.19	2.54	3.60	24.02	36.52	3.42	18.07	1.18	0.04	3.43	2.00	63.48
**MYR**	3.79	0.55	9.61	8.41	6.36	37.33	13.36	5.68	0.20	7.60	7.10	62.67
**SGD**	7.31	3.25	6.06	17.78	15.84	7.15	31.95	2.37	0.12	5.33	2.85	68.05
**PHP**	1.96	0.52	8.31	4.89	3.96	8.20	7.50	53.87	0.11	6.56	4.11	46.13
**VND**	1.20	0.25	0.94	0.91	0.92	0.73	1.01	0.56	92.27	0.38	0.82	7.73
**THB**	4.01	1.76	7.21	7.79	6.88	8.47	10.72	4.96	0.09	42.09	6.01	57.91
**IDR**	3.62	0.51	6.74	6.85	4.71	9.53	7.47	3.70	0.34	7.37	49.16	50.84
**TO**	39.07	12.64	51.76	92.88	81.96	55.20	110.97	27.33	1.30	46.57	32.73	552.40
**NET**	-7.32	-7.65	-11.50	27.21	18.48	-7.47	42.91	-18.79	-6.43	-11.34	-18.11	**TSI = 50.22**

Note:(i) Results are based time-domain model with lag length (*p*) of 1 day and forecasting horizon (*H*) of 100 days.(ii) TSI is the total spillover index in the whole network.TO is the total directional spillover transmitting to the remaining currencies. FROM is the total directional spillovers received from all other currencies. NET is the difference between TO and FROM.

**Table 5:** Static spillover effect between CNH and currencies of RCEP members in frequency domain.

	CNH	JPY	KRW	AUD	NZD	MYR	SGD	PHP	VND	THB	IDR	FROM
**Panel A. Short-term spillover (1–5 days)**												
**CNH**	43.57	0.72	3.37	7.47	6.08	3.08	9.76	1.02	0.17	3.41	1.88	36.97
**JPY**	1.09	64.79	0.41	2.83	4.50	0.08	6.57	0.05	0.03	0.72	0.20	16.48
**KRW**	3.24	0.54	29.47	6.63	5.39	7.36	9.44	4.52	0.11	5.06	4.00	46.28
**AUD**	4.86	1.22	3.59	27.86	18.67	3.31	15.51	1.15	0.02	3.04	2.07	53.44
**NZD**	4.12	2.05	2.90	19.39	29.89	2.77	14.59	0.97	0.03	2.72	1.62	51.17
**MYR**	2.60	0.41	7.21	5.77	4.47	29.33	9.20	4.33	0.14	5.86	5.55	45.55
**SGD**	5.97	2.52	4.99	14.58	13.19	5.98	26.32	1.97	0.11	4.40	2.40	56.09
**PHP**	1.42	0.40	6.76	3.54	2.93	6.45	5.42	44.50	0.09	5.12	3.29	35.41
**VND**	0.70	0.16	0.58	0.58	0.58	0.43	0.54	0.34	73.91	0.22	0.50	4.62
**THB**	2.84	1.18	5.49	5.30	4.72	6.61	7.37	3.99	0.07	32.82	4.70	42.29
**IDR**	2.28	0.33	4.88	4.38	3.10	6.99	4.87	2.81	0.21	5.28	37.11	35.12
**TO**	29.10	9.54	40.17	70.48	63.62	43.07	83.26	21.15	0.98	35.84	26.20	423.40
**NET**	-7.87	-6.95	-6.11	17.04	12.45	-2.48	27.17	-14.25	-3.64	-6.44	-8.92	**TSI = 38.49**
**Panel B. Long-term spillover (more than 5 days)**												
**CNH**	10.04	0.24	0.92	1.87	1.52	0.72	2.56	0.35	0.05	0.90	0.29	9.42
**JPY**	0.34	14.93	0.07	0.64	1.04	0.03	1.44	0.01	0.00	0.19	0.04	3.80
**KRW**	1.35	0.25	7.27	2.78	2.13	2.29	3.96	1.45	0.01	1.83	0.93	16.98
**AUD**	1.11	0.27	0.93	6.47	3.95	0.84	3.62	0.32	0.00	0.75	0.44	12.22
**NZD**	1.07	0.49	0.70	4.63	6.63	0.65	3.48	0.21	0.01	0.71	0.38	12.31
**MYR**	1.19	0.14	2.40	2.64	1.90	8.00	4.16	1.35	0.06	1.74	1.55	17.13
**SGD**	1.35	0.73	1.07	3.20	2.65	1.16	5.63	0.40	0.01	0.93	0.46	11.96
**PHP**	0.55	0.12	1.55	1.35	1.03	1.75	2.08	9.37	0.02	1.44	0.82	10.72
**VND**	0.50	0.09	0.36	0.33	0.34	0.30	0.46	0.22	18.37	0.17	0.32	3.10
**THB**	1.17	0.59	1.72	2.49	2.16	1.85	3.35	0.97	0.02	9.27	1.31	15.62
**IDR**	1.34	0.18	1.87	2.47	1.61	2.54	2.61	0.89	0.13	2.08	12.05	15.73
**To**	9.97	3.10	11.59	22.40	18.34	12.13	27.71	6.18	0.31	10.73	6.53	129.00
**Net**	0.56	-0.70	-5.39	10.18	6.03	-4.99	15.74	-4.54	-2.79	-4.89	-9.20	**TSI = 11.73**

Note: (i) Results are based on the frequency-domain model with lag length (*p*) of 1 day and forecasting horizon (*H*) of 100 days. (ii) TSI is the total spillover index in the whole network.TO is the total directional spillover transmitting to the remaining currencies. FROM is the total directional spillovers received from all other currencies. NET is the difference between TO and FROM.

Similar to the results of CNY, it is apparent from Table 4 that all the currencies are jointly significant to affect each other in this network. More specifically, 50.22% of the forecast error variance of variables in this network can be attributed to the interaction in the network, and 49.78% can be attributed to its own shocks. Table 5 further exhibits the total spillover effect is also dominated by the short term (38.49%), followed by the long term (11.73%).

Upon analyzing the net total directional spillovers, it is evident that SGD, AUD, and NZD remain the primary net transmitters, with values of 42.91%, 27.21%, and 18.48%, respectively. These values exceed those of the CNY network, indicating that other currencies, such as KRW, MYR, and PHP, receive more significant net spillovers. However, the inverse holds for CNH.

As indicated in Table 4, CNH experiences relatively modest net spillover effects from other currencies compared to CNY. Moreover, CNH assumes the role of a net transmitter to a wider range of currencies, namely JPY, KRW, PHP, VND, and IDR, in contrast to CNY. Remarkably, we observe that the other currencies maintain a consistent role, either as net transmitters or net receivers, across all frequency bands, while CNH operates as a net transmitter over the long term, in contrast to the short term. This reinforces the finding that the RMB exerts a more substantial influence over the long term.

The aforementioned findings indicate that CNH may wield a greater influence than CNY, aligning with the findings of Zhou et al. [47] and Chow [48]. This can be attributed to the offshore market’s heightened flexibility, the greater diversification of market participants, and the broader product range that contribute to its pricing power [67, 68]. Additionally, both CNH and CNY exhibit a more substantial impact in the long term, primarily due to the influence generated by renminbi through trade and financial linkages [11, 48].

#### 3.1.3 further analysis

In the study above, our objective was to ascertain the mean spillover effect between RMB and currencies of RCEP members. However, the RMB exchange rate regime reform implemented on 11 August 2015 ("8.11" RMB exchange rate regime reform), has significantly influenced both CNY and CNH [69]. As a result, we conduct further analysis to examine the spillover effect during the period after the "8.11" RMB exchange rate regime reform, from 11 August 2015 to 19 August 2022.

Based on Tables 6 and 7, it is evident that the total spillover effects exhibit greater strength subsequent to the "8.11" RMB exchange rate regime reform, in comparison to the full sample period. The net total directional spillovers for CNY and CNH rank fifth and fourth respectively, both surpassing the figures for the full sample period, with CNH functioning as a net transmitter. Specifically for the frequency bands, in the long term, both CNH and CNY act as net transmitters, with CNH even surpassing NZD in the ranking. This implies that in the immediate future, RMB is still "under constraint", and its impact is mainly realized through long-term channels, including international trade and foreign direct investment. Thus, it is unmistakable that the "8.11" RMB exchange rate regime reform constitutes a significant point in time that exerted a momentous influence on the spillover effect of RMB.

**Table 6:** Static spillover effect between CNY and currencies of RCEP members in time-frequency domain: The period after the "8.11" RMB exchange rate regime reform.

	CNY	JPY	KRW	AUD	NZD	MYR	SGD	PHP	VND	THB	IDR	FROM
**Panel A. Overall spillover (time domain)**												
**CNY**	45.18	1.27	6.32	8.31	7.14	4.88	12.11	2.09	0.83	7.17	4.70	54.82
**JPY**	2.25	76.98	0.37	2.82	6.47	0.16	8.45	0.17	0.12	1.69	0.52	23.02
**KRW**	5.50	0.61	35.49	8.87	6.92	9.80	13.10	4.25	0.32	8.02	7.12	64.51
**AUD**	5.81	1.17	4.79	31.93	21.48	4.74	18.33	0.96	0.14	5.06	5.60	68.07
**NZD**	5.22	2.80	3.92	22.35	33.08	4.12	17.95	0.93	0.21	4.61	4.80	66.92
**MYR**	4.45	0.57	9.97	7.82	6.14	36.30	12.53	3.77	1.87	6.97	9.60	63.70
**SGD**	7.62	3.22	6.32	16.66	15.67	7.08	29.04	1.80	0.45	6.74	5.40	70.96
**PHP**	2.90	0.92	6.25	3.53	3.32	5.65	5.96	59.79	0.77	5.54	5.37	40.21
**VND**	2.76	0.34	1.69	2.12	1.24	2.87	2.63	1.24	79.06	1.40	4.64	20.94
**THB**	6.07	2.58	7.81	8.80	7.34	7.29	12.49	3.39	0.58	37.49	6.15	62.51
**IDR**	4.27	0.75	7.65	10.01	7.66	10.19	9.86	3.39	2.40	6.14	37.68	62.32
**To**	46.87	14.23	55.08	91.30	83.38	56.78	113.41	21.99	7.71	53.34	53.90	597.98
**Net**	-7.95	-8.79	-9.43	23.23	16.46	-6.92	42.45	-18.22	-13.24	-9.16	-8.43	**TSI = 54.36**
**Panel B. Short-term spillover (1–5 days)**												
**CNY**	36.74	0.99	4.98	6.51	5.68	3.82	9.29	1.53	0.64	5.65	3.94	43.03
**JPY**	1.68	62.75	0.28	2.20	5.22	0.10	6.62	0.12	0.12	1.18	0.44	17.98
**KRW**	4.04	0.41	28.12	6.13	4.84	7.39	9.03	3.07	0.26	5.84	5.71	46.73
**AUD**	4.78	0.96	3.66	25.50	17.37	3.59	14.51	0.71	0.12	4.07	4.50	54.26
**NZD**	4.13	2.20	3.01	17.67	26.68	3.27	14.09	0.74	0.17	3.64	3.83	52.73
**MYR**	3.01	0.41	7.07	5.02	4.07	27.18	8.13	2.53	1.20	5.09	7.07	43.59
**SGD**	6.17	2.49	4.99	13.31	12.74	5.74	23.39	1.44	0.35	5.44	4.37	57.04
**PHP**	2.09	0.75	5.03	2.46	2.35	4.33	4.15	48.43	0.53	4.28	4.28	30.24
**VND**	1.74	0.22	1.03	1.32	0.73	1.88	1.53	0.73	63.62	0.88	3.01	13.07
**THB**	4.48	1.68	5.64	5.68	4.84	5.26	8.16	2.55	0.40	28.45	4.45	43.14
**IDR**	2.83	0.47	5.23	5.98	4.72	6.89	6.03	2.35	1.46	4.23	26.73	40.18
**TO**	34.94	10.58	40.91	66.29	62.56	42.26	81.54	15.76	5.24	40.29	41.61	441.99
**NET**	-8.09	-7.39	-5.81	12.03	9.83	-1.33	24.50	-14.48	-7.83	-2.85	1.43	**TSI = 40.18**
**Panel C. Long-term spillover (more than 5 days)**												
**CNY**	8.44	0.28	1.34	1.80	1.45	1.06	2.82	0.56	0.19	1.51	0.77	11.79
**JPY**	0.57	14.23	0.09	0.62	1.24	0.06	1.82	0.04	0.00	0.51	0.08	5.04
**KRW**	1.46	0.20	7.36	2.74	2.08	2.41	4.08	1.18	0.06	2.18	1.40	17.79
**AUD**	1.03	0.20	1.13	6.43	4.11	1.14	3.82	0.26	0.03	0.99	1.10	13.82
**NZD**	1.09	0.61	0.92	4.68	6.41	0.86	3.86	0.19	0.05	0.97	0.97	14.18
**MYR**	1.44	0.16	2.90	2.80	2.08	9.12	4.40	1.24	0.68	1.88	2.54	20.10
**SGD**	1.46	0.72	1.33	3.35	2.93	1.35	5.65	0.36	0.10	1.30	1.02	13.91
**PHP**	0.82	0.16	1.22	1.07	0.98	1.32	1.81	11.36	0.25	1.26	1.09	9.97
**VND**	1.02	0.12	0.65	0.80	0.52	0.99	1.10	0.51	15.43	0.52	1.62	7.87
**THB**	1.60	0.90	2.16	3.12	2.50	2.03	4.34	0.85	0.17	9.04	1.70	19.37
**IDR**	1.44	0.29	2.42	4.04	2.94	3.30	3.82	1.05	0.94	1.91	10.94	22.14
**TO**	11.93	3.64	14.17	25.01	20.82	14.52	31.87	6.23	2.46	13.05	12.28	155.98
**NET**	0.14	-1.40	-3.62	11.20	6.63	-5.59	17.95	-3.74	-5.41	-6.31	-9.86	**TSI = 14.18**

Note: (i) Results are based on the time-domain and frequency-domain model with lag length (*p*) of 1 day and forecasting horizon (*H*) of 100 days. (ii) TSI is the total spillover index in the whole network.TO is the total directional spillover transmitting to the remaining currencies. FROM is the total directional spillovers received from all other currencies. NET is the difference between TO and FROM.

**Table 7:** Static spillover effect between CNH and currencies of RCEP members in time-frequency domain: The period after the "8.11" RMB exchange rate regime reform.

	CNH	JPY	KRW	AUD	NZD	MYR	SGD	PHP	VND	THB	IDR	FROM
**Panel A. Overall spillover (time domain)**												
**CNH**	43.93	2.44	4.10	9.80	8.63	4.31	16.08	1.53	1.35	4.96	2.86	56.07
**JPY**	3.24	69.69	1.27	3.50	6.10	1.04	8.98	1.20	1.42	2.40	1.14	30.31
**KRW**	5.89	1.19	34.59	8.55	6.54	9.74	13.03	5.06	0.71	7.71	6.99	65.41
**AUD**	7.65	1.51	4.07	33.33	20.26	4.10	18.16	1.58	0.81	4.50	4.03	66.67
**NZD**	6.89	3.08	3.38	21.29	34.95	3.59	17.25	1.27	0.57	4.08	3.65	65.05
**MYR**	5.82	1.25	9.64	7.04	5.55	34.68	11.42	4.86	1.30	8.80	9.66	65.32
**SGD**	10.98	3.89	5.57	15.82	14.42	6.37	28.95	2.25	0.68	6.59	4.49	71.05
**PHP**	2.74	1.58	6.71	3.98	3.46	6.30	5.93	54.94	1.23	5.92	7.21	45.06
**VND**	4.05	1.76	1.53	1.59	1.04	1.83	2.70	1.62	78.93	2.27	2.68	21.07
**THB**	5.31	3.32	7.24	7.56	6.47	8.84	12.47	4.29	1.03	36.91	6.57	63.09
**IDR**	4.31	1.29	7.32	7.74	6.16	10.58	9.35	5.56	1.70	7.26	38.74	61.26
**TO**	56.88	21.31	50.83	86.86	78.64	56.70	115.37	29.22	10.79	54.50	49.28	610.37
**NET**	0.81	-9.00	-14.58	20.19	13.59	-8.62	44.31	-15.84	-10.28	-8.59	-11.98	**TSI = 55.49**
**Panel B. Short-term spillover (1–5 days)**												
**CNH**	35.16	1.94	3.26	8.23	7.28	3.43	13.13	1.18	1.08	3.97	2.40	45.92
**JPY**	2.55	56.14	1.02	2.89	5.18	0.77	7.47	0.98	1.05	1.82	0.99	24.72
**KRW**	4.07	0.85	27.48	6.09	4.73	7.32	9.20	3.67	0.56	5.58	5.52	47.57
**AUD**	6.14	1.16	3.11	26.72	16.39	3.09	14.33	1.19	0.64	3.54	3.21	52.79
**NZD**	5.43	2.37	2.63	17.06	28.41	2.76	13.67	1.01	0.45	3.18	2.92	51.48
**MYR**	3.66	0.82	6.95	4.55	3.70	25.86	7.41	3.39	0.86	6.33	7.13	44.80
**SGD**	8.76	2.98	4.45	12.83	11.83	5.04	23.51	1.82	0.55	5.27	3.71	57.23
**PHP**	2.03	1.37	5.56	3.00	2.64	5.06	4.38	44.16	0.96	4.69	5.85	35.53
**VND**	2.56	1.20	1.02	1.14	0.72	1.11	1.63	1.07	62.80	1.47	1.64	13.56
**THB**	3.65	2.10	5.26	5.02	4.35	6.51	8.27	3.29	0.74	27.77	5.07	44.25
**IDR**	2.75	0.84	5.45	5.00	4.06	7.73	6.09	4.08	1.11	5.19	28.78	42.30
**TO**	41.60	15.62	38.73	65.81	60.87	42.80	85.58	21.67	8.01	41.03	38.44	460.15
**NET**	-4.31	-9.10	-8.85	13.02	9.39	-1.99	28.34	-13.87	-5.55	-3.22	-3.86	**TSI = 41.83**
**Panel C. Long-term spillover (more than 5days)**												
**CNH**	8.78	0.50	0.84	1.57	1.35	0.88	2.94	0.35	0.26	0.99	0.46	10.15
**JPY**	0.69	13.55	0.25	0.61	0.92	0.27	1.52	0.23	0.36	0.59	0.15	5.59
**KRW**	1.82	0.34	7.11	2.45	1.82	2.43	3.83	1.39	0.15	2.14	1.47	17.84
**AUD**	1.51	0.35	0.96	6.61	3.87	1.01	3.83	0.39	0.17	0.96	0.82	13.88
**NZD**	1.46	0.72	0.75	4.22	6.54	0.82	3.58	0.27	0.12	0.90	0.74	13.57
**MYR**	2.17	0.43	2.69	2.48	1.86	8.81	4.00	1.47	0.43	2.47	2.53	20.53
**SGD**	2.21	0.92	1.11	2.99	2.59	1.33	5.43	0.43	0.13	1.33	0.78	13.82
**PHP**	0.71	0.21	1.15	0.99	0.81	1.25	1.56	10.77	0.27	1.23	1.36	9.53
**VND**	1.49	0.56	0.51	0.45	0.32	0.72	1.07	0.56	16.13	0.80	1.04	7.52
**THB**	1.66	1.21	1.97	2.54	2.12	2.33	4.20	1.00	0.30	9.14	1.50	18.83
**IDR**	1.55	0.45	1.86	2.74	2.11	2.85	3.26	1.47	0.59	2.07	9.96	18.96
**TO**	15.27	5.70	12.10	21.05	17.77	13.90	29.79	7.55	2.78	13.47	10.84	150.22
**NET**	5.12	0.11	-5.74	7.16	4.20	-6.63	15.97	-1.98	-4.73	-5.37	-8.12	**TSI = 13.66**

Note: (i) Results are based on the time-domain and frequency-domain model with lag length (*p*) of 1 day and forecasting horizon (*H*) of 100 days. (ii) TSI is the total spillover index in the whole network.TO is the total directional spillover transmitting to the remaining currencies. FROM is the total directional spillovers received from all other currencies. NET is the difference between TO and FROM.

Previous studies have highlighted that market turmoil triggered by significant economic and political events can affect the transmission of spillovers across foreign exchange markets [5]. As such, the static analysis presents a somewhat limited perspective, disregarding the influence of significant political and economic occurrences throughout the entire time frame under examination. To obtain a more comprehensive understanding, we need to examine the time-varying spillover effects.

### 3.2. Time-varying spillover effects

We employ the TVP-VAR model to investigate the dynamic spillover effects between RMB (CNY and CNH, respectively) and currencies of RCEP members, encompassing both the time and frequency domains.

#### 3.2.1 Time-varying spillover effect between CNY and currencies of RCEP members

**Fig 1:**
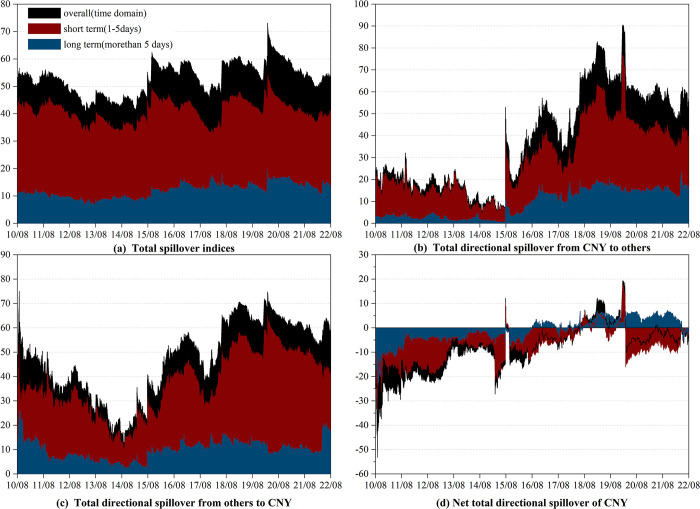
Time-varying spillover between CNY and currencies of RCEP members in time-frequency domain. (i) Results are based on the time-domain and frequency-domain model with lag length (*p*) of 1 day and forecasting horizon *(H*) of 100 days (ii) "overall" refers to the spillover observed in the time domain, which is represented by the black shade. (iii) "short term" and "long term" refer to the spillovers observed in the high and low frequency bands, respectively, which are represented by the red and blue shades.

The outcomes of the correlational examination are presented in Fig 1, exhibiting the dynamic evolution of not only the overall spillovers (highlighted by the black shade) but also the short-term (highlighted by the red shade) and long-term (highlighted by the blue shade) spillovers.

We begin our analysis with the time-varying total spillover effect, which reflects the dynamic connectedness among currency markets over the entire sample period. As depicted in Fig 1A, the overall total spillover ranges from 40.36% to 73.10%, reinforcing our preliminary conclusion that the whole currency system is highly interconnected. Notably, we observe certain spikes during specific periods, which are closely tied to significant events [19]. Specifically, the overall total spillover declined following the European debt crisis but peaked between August and November 2015 due to the “8.11” RMB exchange rate regime reform. The overall total spillover increased again in June 2016 due to the Brexit referendum and was further boosted by the outbreak of the China-US trade war in 2018. The COVID-19 pandemic pushed the overall total spillover to its highest point. The most recent increase occurred after the outbreak of the Russia-Ukraine conflict in February 2022. Surprisingly, we do not observe a significant effect of the RCEP signing or taking effect on the total spillover. One possible explanation for this finding is that RCEP primarily fosters trade collaboration among nations in the area and has yet to exert a substantial impact due to the repercussions of the COVID-19 pandemic. Furthermore, the overall and short-term total spillover exhibit similar evolution trends, and the short-term total spillover is much higher than the long-term total spillover, indicating that the total spillovers between currencies mainly occur in the short term. This also suggests that the various markets process relevant information quickly.

Next, we focus on the role of CNY in the whole currency market. The relevant findings are illustrated in Fig 1B to 1d, which demonstrate the total directional spillover from CNY to other currencies (CNY_*To*_), total directional spillover from other currencies to CNY (CNY_*From*_), and net total directional spillover of CNY (CNY_*Net*_), respectively. It is important to note that CNY_*Net*_ represents the difference between CNY_*To*_ and CNY_*From*_, indicating that CNY is a net transmitter(receiver) when CNY_*Net*_ is positive(negative).

Similar to the total spillover, CNY_*To*_ and CNY_*From*_ are highly event-dependent and vary over time. Notably, the "8.11" RMB exchange rate regime reform boosted CNY_*To*_ sharply, particularly in the short term. Zhou et al. arrived at the similar conclusion in their study on the influence of the Renminbi in countries along the Belt and Road [47]. It is worth mentioning that CNY_*To*_ is more sensitive to events than CNY_*From*_. For instance, the "8.11" RMB exchange rate regime reform, the China-US trade war, and the COVID-19 pandemic have had a more significant impact on CNY_*To*_ than on CNY_*From*_. Due to the absence of a freely floating exchange rate regime and a fully open capital account, CNY is less vulnerable to shocks from other currencies. This implies that the CNY has a greater impact on other currencies during significant event shocks, while also possessing the capability to withstand the repercussions of shocks to other currencies.

Regarding CNY_*Net*_, the time-varying results depicted in Fig 1D very interesting. Until August 2015, CNY was a net receiver with short-term connectedness prevailing. After the "8.11" RMB exchange rate regime reform, CNY shifted to being a net transmitter for a relatively short period, after which it reverted to its previous position as a net receiver. However, it should be noted that CNY played a considerable role as a net transmitter between June 2018 and March 2020, which was associated with the impact of the China-US trade war and the COVID-19 pandemic. In terms of the frequency domain, long-term net total directional spillover was predominant from the end of 2018 to the end of 2019, implying that changes in the market itself triggered by the China-US trade war were reflected in the evolution of spillovers in the long term. In contrast, the onset of the COVID-19 pandemic caused a short-term shock mainly through the channels of confidence and expectation. Although CNY has gradually transitioned to a net transmitter since August 2015, it has been mainly due to positive long-term net spillovers. In the short term, it has still primarily been a net receiver.

In conclusion, we posit that the net transmission position of CNY has undergone significant improvement post the "8.11" RMB exchange rate regime reform, shifting from a pure net receiver to an alternating net receiver and net transmitter. Moreover, the influence of CNY is sensitivity to major events. Certain events may amplify the short-term impact of CNY, while others may contribute to long-term influence.

#### 3.2.2 Time-varying spillover effect between CNH and currencies of RCEP members

**Fig 2:**
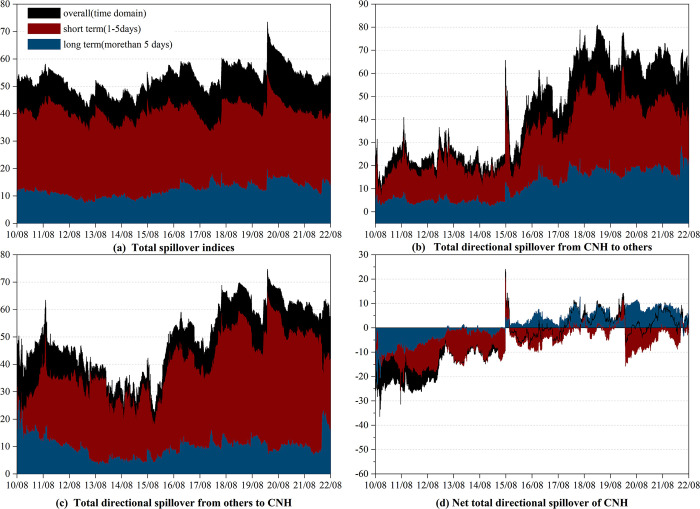
Time-varying spillover between CNH and currencies of RCEP members in time-frequency domain. (i) Results are based on the time-domain and frequency-domain model with lag length (*p*) of 1 day and forecasting horizon (*H*) of 100 days (ii) "overall" refers to the spillover observed in the time domain, which is represented by the black shade. (iii) "short term" and "long term" refer to the spillovers observed in the high and low frequency bands, respectively, which are represented by the red and blue shades.

As can be seen from Fig 2A, the overall total spillover between CNH and RCEP currencies exhibits time-varying characteristics, fluctuating between 40.83% to 73.52%. Similar to the total spillover between CNY and currencies of RCEP members, it is driven by short-term connectedness and sensitive to the international economic and political events. For example, we observe spikes during the “8.11” RMB exchange rate regime reform, the Brexit referendum, the China-US trade war, the COVID-19 pandemic, and the Russia-Ukraine conflict. Notably, the spillover peaked at unprecedented levels during the initial stages of the COVID-19 pandemic. However, our findings indicate that the total spillover between the CNH and RCEP currencies differs from that of the CNY and RCEP currencies in specific aspects. The former shows a weaker reaction to the “8.11” RMB exchange rate regime reform. This could be attributed to the fact that the "8.11" RMB exchange rate regime reform has enhanced the central parity formation mechanism of the RMB exchange rate, thereby improving the exchange rate flexibility of the CNY[69].

We now turn to the spillover effect of CNH. Fig 2B to 2D represent the total directional spillover from CNH to other currencies (CNH_*To*_), total directional spillover from other currencies to CNH (CNH_*From*_), and net total directional spillover of CNH (CNH_*Net*_), respectively. It should also be noted that CNH_*Net*_ equals to CNH_*To*_ minus CNH_*From*_. When it is positive (negative), CNH acts as a net transmitter(receiver).

Firstly, as displayed in Fig 2B, CNH_*To*_ and CNY_*To*_ exhibit similar patterns, both oscillating upwards and being more prevalent in the short term. Nonetheless, before August 2015, CNH_*To*_ exerted a stronger influence than the CNY_*To*_, indicating the greater market influence of the CNH. While the “8.11” RMB exchange rate regime reform has improved both CNH_*To*_ and CNY_*To*_, the latter experienced a greater increase. In specifics, CNY_*To*_ at the end of August was approximately five times higher than at the beginning of August, while CNH_*To*_ only doubled. More interestingly, COVID-19 and the China-US trade war also had a greater impact on the growth rate of CNY_*To*_.

Secondly, from 2014 to August 2015, CNH_*From*_ was higher than CNY_*From*_ due to more price elastic of CNH. Throughout the entire sample period, consistent with CNY, CNH_*From*_ is less affected by events than CNH_*To*_, which demonstrates that CNH has a high capacity to resist market risk.

Finally, let us proceed to the dynamic evolution of CNH_*Net*_ illustrated in Fig 2D. Like CNY_*Net*_, CNH_*Net*_ was negative on all frequency bands until the “8.11” RMB exchange rate regime reform, meaning that CNH assumed a considerable role as a net receiver. The “8.11” RMB exchange rate regime reform also caused a short peak in the CNH_*Net*_, which subsequently returned to net receiver status until the end of 2017. Since then, CNH has switched between being a net receiver and a net transmitter, with the duration of the net transmitter being longer than that of CNY. Specifically, we note that CNH remains a net transmitter from the end of 2017 to the end of March 2020 and again from February 2021 to August 2022 (excluding some short periods). Long-term connectedness dominated in both of the above intervals, except for early 2020. As already mentioned, the first interval was marked by the China-US trade war and the COVID-19 pandemic, while the second interval was characterized by the promotion of vaccines in China.

In brief, following the "8.11" RMB exchange rate regime reform, the CNH emerged as a net transmitter, particularly over the long term. The net transmitter status of the CNH has been bolstered by events such as the COVID-19 pandemic and the China-US trade war. Notably, CNH wields greater influence than the CNY. However, in times of significant market turbulence, compared to CNH, the directional spillover of CNY to the currencies of RCEP members exhibits a more significant ascent. The reason for this could be attributed to the relatively shallow liquidity base that constrains the offshore RMB market [11].

### 3.3 Robustness analysis

In this section, we conduct the several research tests to ensure the robustness of our empirical results.

Firstly, we validate the static spillover effects using the average results of the TVP-VAR model, as shown in Tables 8 and 9. In both cases, the total spillover effects exceed 50%, dominated by short-term connectedness. Further, the influence of the RMB presents the frequency band and country heterogeneity. In general, the influence of CNH is greater than that of CNY. These results are consistent with the empirical results in section 3.1, further confirming the robustness of our conclusions.

**Table 8:** Average spillover effect between CNY and currencies of RCEP members in time-frequency domain.

	CNY	JPY	KRW	AUD	NZD	MYR	SGD	PHP	VND	THB	IDR	FROM
**Panel A. Overall spillover (time domain)**												
**CNY**	53.90	1.79	4.91	5.57	4.88	6.23	8.99	2.94	1.08	5.77	3.95	46.10
**JPY**	2.09	73.05	1.53	3.90	5.56	1.31	8.14	0.99	1.05	1.45	0.92	26.95
**KRW**	4.10	1.41	36.16	8.47	7.02	9.92	12.41	6.62	0.72	7.30	5.86	63.84
**AUD**	3.30	2.06	4.61	34.44	21.87	4.87	19.15	2.05	0.47	4.02	3.16	65.56
**NZD**	3.08	2.99	3.77	23.33	36.73	4.00	17.55	1.58	0.44	3.70	2.84	63.27
**MYR**	4.81	1.17	9.30	7.92	6.34	34.44	12.48	6.23	0.81	8.39	8.11	65.56
**SGD**	5.17	3.83	6.25	17.35	14.96	7.89	31.22	2.87	0.51	6.18	3.78	68.78
**PHP**	2.83	1.11	8.11	5.03	4.06	8.19	7.02	50.51	0.71	6.02	6.41	49.49
**VND**	2.52	1.25	1.55	1.21	1.16	1.60	1.65	1.12	84.04	1.43	2.47	15.96
**THB**	4.93	2.07	7.30	6.98	6.37	9.48	10.66	5.07	0.75	39.91	6.47	60.09
**IDR**	3.62	0.88	6.76	5.98	4.73	9.88	7.41	5.49	1.22	7.25	46.78	53.22
**TO**	36.43	18.56	54.07	85.75	76.96	63.38	105.46	34.97	7.76	51.52	43.97	578.82
**NET**	-9.67	-8.39	-9.77	20.20	13.68	-2.18	36.68	-14.52	-8.20	-8.57	-9.25	**TSI = 52.62**
**Panel B. Short-term spillover (1-5days)**												
**CNY**	43.36	1.36	3.87	4.33	3.86	5.05	6.86	2.31	0.90	4.63	3.26	36.42
**JPY**	1.65	59.16	1.23	3.28	4.65	1.00	6.81	0.83	0.81	1.09	0.79	22.15
**KRW**	3.13	1.03	29.04	6.11	5.17	7.58	8.86	5.10	0.63	5.43	4.81	47.85
**AUD**	2.72	1.69	3.73	28.15	18.06	3.89	15.75	1.65	0.39	3.20	2.58	53.66
**NZD**	2.47	2.43	3.07	19.04	30.13	3.22	14.43	1.32	0.37	2.93	2.32	51.60
**MYR**	3.44	0.83	7.03	5.54	4.54	26.73	8.69	4.79	0.58	6.43	6.27	48.16
**SGD**	4.22	2.99	5.15	14.34	12.51	6.46	25.82	2.41	0.44	5.06	3.18	56.77
**PHP**	2.24	0.95	6.68	3.77	3.12	6.52	5.20	41.24	0.55	4.65	5.20	38.89
**VND**	1.67	0.88	1.07	0.90	0.86	1.12	1.08	0.80	68.54	0.99	1.75	11.13
**THB**	3.90	1.40	5.70	4.85	4.47	7.32	7.48	4.03	0.60	30.95	5.16	44.90
**IDR**	2.61	0.61	5.18	4.06	3.30	7.33	5.07	4.23	0.88	5.36	36.52	38.62
**TO**	28.06	14.17	42.70	66.24	60.55	49.48	80.22	27.46	6.15	39.77	35.33	450.13
**NET**	-8.35	-7.97	-5.14	12.58	8.95	1.32	23.45	-11.43	-4.98	-5.13	-3.29	**TSI = 40.92**
**Panel C. Long-term spillover (more than 5days)**												
**CNY**	10.54	0.43	1.04	1.24	1.02	1.18	2.13	0.63	0.18	1.14	0.70	9.68
**JPY**	0.44	13.89	0.30	0.62	0.91	0.32	1.33	0.16	0.23	0.36	0.13	4.80
**KRW**	0.97	0.38	7.12	2.36	1.85	2.35	3.55	1.53	0.10	1.87	1.04	15.99
**AUD**	0.58	0.36	0.88	6.29	3.80	0.98	3.41	0.40	0.08	0.83	0.58	11.90
**NZD**	0.61	0.56	0.70	4.28	6.60	0.78	3.12	0.26	0.07	0.77	0.52	11.67
**MYR**	1.37	0.33	2.26	2.38	1.80	7.71	3.80	1.44	0.23	1.96	1.83	17.41
**SGD**	0.95	0.83	1.10	3.01	2.46	1.43	5.40	0.46	0.07	1.11	0.59	12.01
**PHP**	0.58	0.17	1.43	1.26	0.94	1.68	1.82	9.27	0.16	1.37	1.20	10.61
**VND**	0.85	0.37	0.49	0.31	0.30	0.48	0.56	0.32	15.50	0.43	0.72	4.83
**THB**	1.03	0.68	1.60	2.12	1.90	2.16	3.18	1.05	0.16	8.96	1.31	15.18
**IDR**	1.01	0.27	1.58	1.92	1.44	2.55	2.34	1.26	0.34	1.90	10.26	14.60
**TO**	8.36	4.38	11.37	19.51	16.41	13.91	25.24	7.51	1.61	11.74	8.63	128.69
**NET**	-1.32	-0.42	-4.62	7.61	4.73	-3.50	13.23	-3.09	-3.22	-3.44	-5.96	**TSI = 11.70**

Note: (i) Results are based on the time-domain and frequency-domain model with lag length (*p*) of 1 day and forecasting horizon (H) of 100 days. (ii) TSI is the total spillover index in the whole network.TO is the total directional spillover transmitting to the remaining currencies. FROM is the total directional spillovers received from all other currencies. NET is the difference between TO and FROM.

**Table 9:** Average spillover effect between CNH and currencies of RCEP members in time-frequency domain.

	CNH	JPY	KRW	AUD	NZD	MYR	SGD	PHP	VND	THB	IDR	FROM
**Panel A. Overall spillover (time domain)**												
**CNH**	51.32	1.97	3.83	8.54	7.35	4.77	12.63	2.04	0.79	4.17	2.59	48.68
**JPY**	2.29	72.54	1.48	4.00	5.71	1.10	8.60	0.99	0.90	1.53	0.87	27.46
**KRW**	4.38	1.55	36.23	8.51	6.92	9.91	12.30	6.74	0.62	7.01	5.85	63.77
**AUD**	5.50	2.02	4.42	34.38	21.52	4.41	18.77	1.88	0.40	3.87	2.83	65.62
**NZD**	5.00	3.02	3.55	23.00	36.83	3.60	17.25	1.50	0.36	3.44	2.45	63.17
**MYR**	4.72	1.15	9.41	7.86	6.23	34.71	12.47	6.19	0.62	8.48	8.15	65.29
**SGD**	7.79	4.11	5.92	16.95	14.64	7.35	31.00	2.64	0.42	5.78	3.39	69.00
**PHP**	2.44	1.15	8.42	4.79	3.86	8.19	6.78	50.87	0.68	6.37	6.45	49.13
**VND**	2.54	1.12	1.30	1.20	1.12	1.21	1.61	1.05	85.94	1.25	1.67	14.06
**THB**	4.08	2.25	7.11	7.01	6.39	9.50	10.67	5.39	0.65	40.47	6.49	59.53
**IDR**	3.33	0.89	6.84	5.91	4.53	9.85	7.41	5.61	0.85	7.48	47.30	52.70
**TO**	42.07	19.23	52.27	87.77	78.26	59.88	108.48	34.04	6.29	49.38	40.74	578.42
**NET**	-6.61	-8.23	-11.50	22.15	15.09	-5.41	39.48	-15.09	-7.77	-10.15	-11.96	**TSI = 52.58**
**Panel B. Short-term spillover (1–5 days)**												
**CNH**	40.47	1.50	3.03	6.88	5.89	3.93	10.14	1.57	0.64	3.34	2.12	39.04
**JPY**	1.78	58.54	1.20	3.32	4.78	0.81	7.16	0.81	0.70	1.18	0.72	22.46
**KRW**	3.12	1.11	29.12	6.10	5.08	7.59	8.75	5.14	0.51	5.19	4.75	47.35
**AUD**	4.47	1.63	3.57	28.03	17.81	3.51	15.34	1.48	0.33	3.05	2.29	53.47
**NZD**	3.98	2.41	2.88	18.66	30.15	2.86	14.04	1.22	0.29	2.71	1.96	51.00
**MYR**	3.19	0.81	7.12	5.48	4.45	26.90	8.63	4.72	0.44	6.45	6.30	47.60
**SGD**	6.34	3.21	4.89	13.94	12.21	6.02	25.55	2.20	0.35	4.72	2.82	56.70
**PHP**	1.86	0.98	6.94	3.57	2.96	6.53	5.01	41.41	0.54	4.95	5.28	38.62
**VND**	1.65	0.76	0.88	0.91	0.83	0.80	1.04	0.72	69.91	0.83	1.07	9.50
**THB**	2.95	1.50	5.52	4.84	4.45	7.40	7.38	4.28	0.49	31.31	5.17	43.98
**IDR**	2.24	0.62	5.22	3.99	3.15	7.41	5.03	4.32	0.59	5.48	36.97	38.05
**TO**	31.58	14.54	41.25	67.69	61.61	46.86	82.52	26.46	4.88	37.90	32.48	447.78
**NET**	-7.46	-7.92	-6.10	14.22	10.61	-0.74	25.83	-12.16	-4.61	-6.09	-5.57	**TSI = 40.71**
**Panel C. Long-term spillover (more than 5 days)**												
**CNH**	10.84	0.47	0.79	1.66	1.46	0.85	2.49	0.46	0.15	0.83	0.47	9.64
**JPY**	0.50	13.99	0.28	0.67	0.94	0.29	1.43	0.18	0.20	0.36	0.15	5.00
**KRW**	1.26	0.43	7.11	2.41	1.84	2.32	3.55	1.60	0.11	1.82	1.10	16.43
**AUD**	1.04	0.40	0.85	6.34	3.71	0.90	3.43	0.41	0.07	0.81	0.54	12.15
**NZD**	1.02	0.61	0.67	4.34	6.68	0.73	3.21	0.28	0.06	0.74	0.49	12.16
**MYR**	1.53	0.34	2.29	2.38	1.78	7.81	3.84	1.47	0.18	2.03	1.84	17.69
**SGD**	1.46	0.90	1.04	3.02	2.43	1.32	5.44	0.45	0.07	1.06	0.57	12.30
**PHP**	0.58	0.17	1.48	1.22	0.90	1.65	1.77	9.46	0.14	1.42	1.17	10.51
**VND**	0.89	0.35	0.41	0.29	0.28	0.41	0.57	0.33	16.04	0.42	0.61	4.56
**THB**	1.13	0.75	1.59	2.17	1.94	2.10	3.28	1.10	0.16	9.16	1.33	15.54
**IDR**	1.09	0.27	1.62	1.92	1.38	2.44	2.38	1.30	0.26	1.99	10.33	14.65
**TO**	10.49	4.69	11.03	20.08	16.65	13.02	25.96	7.58	1.41	11.49	8.26	130.64
**NET**	0.85	-0.31	-5.40	7.93	4.49	-4.67	13.65	-2.93	-3.16	-4.06	-6.39	**TSI = 11.88**

Note: (i) Results are based on the time-domain and frequency-domain model with lag length (*p*) of 1 day and forecasting horizon (H) of 100 days. (ii) TSI is the total spillover index in the whole network.TO is the total directional spillover transmitting to the remaining currencies. FROM is the total directional spillovers received from all other currencies. NET is the difference between TO and FROM.

**Fig 3:**
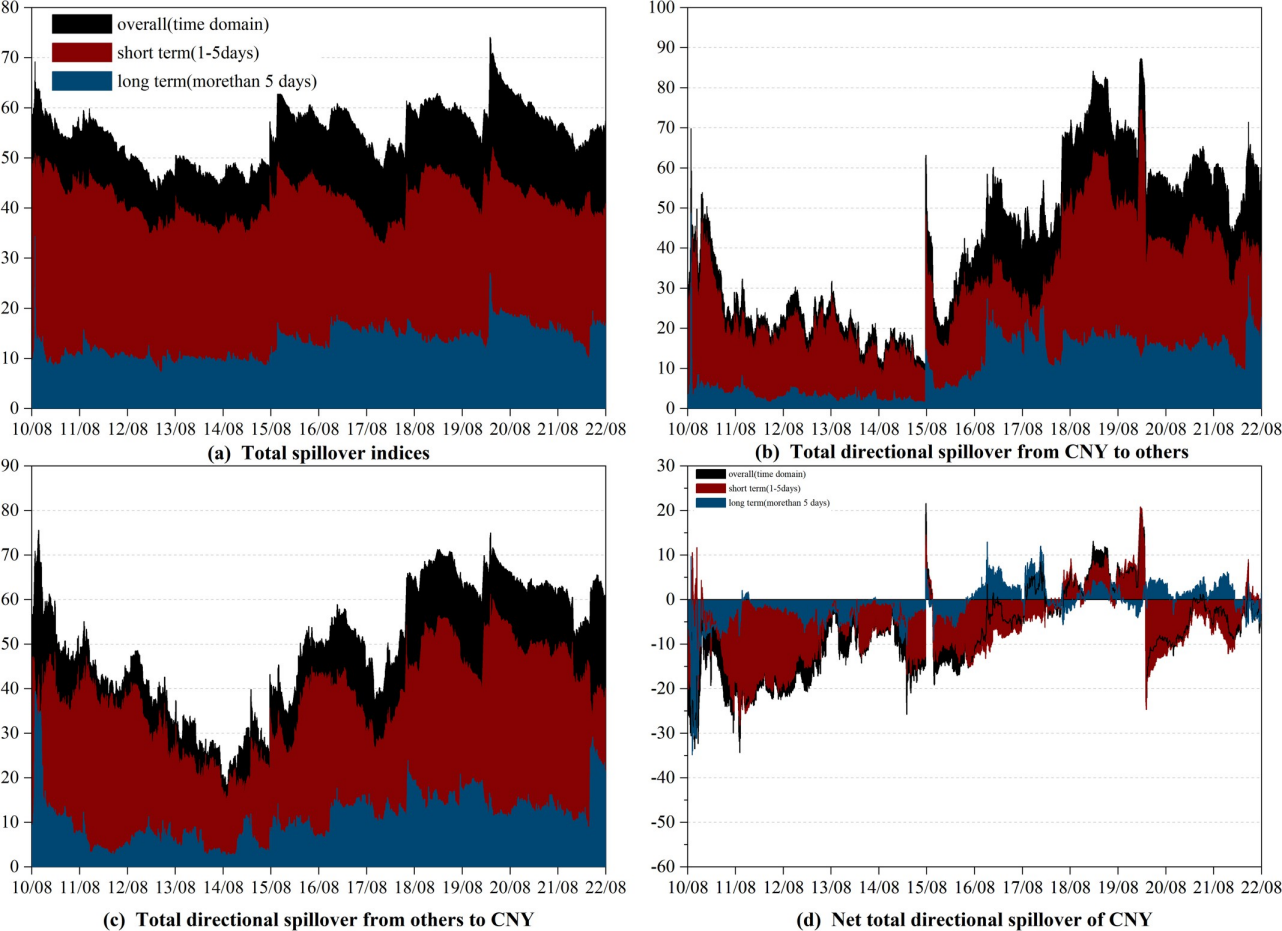
Robust of spillover between CNY and currencies of RCEP members (*p* = 3, *H* = 100). (i) "overall" refers to the spillover observed in the time domain, which is represented by the black shade. (ii) "short term" and "long term" refer to the spillovers observed in the high and low frequency bands.

**Fig 4:**
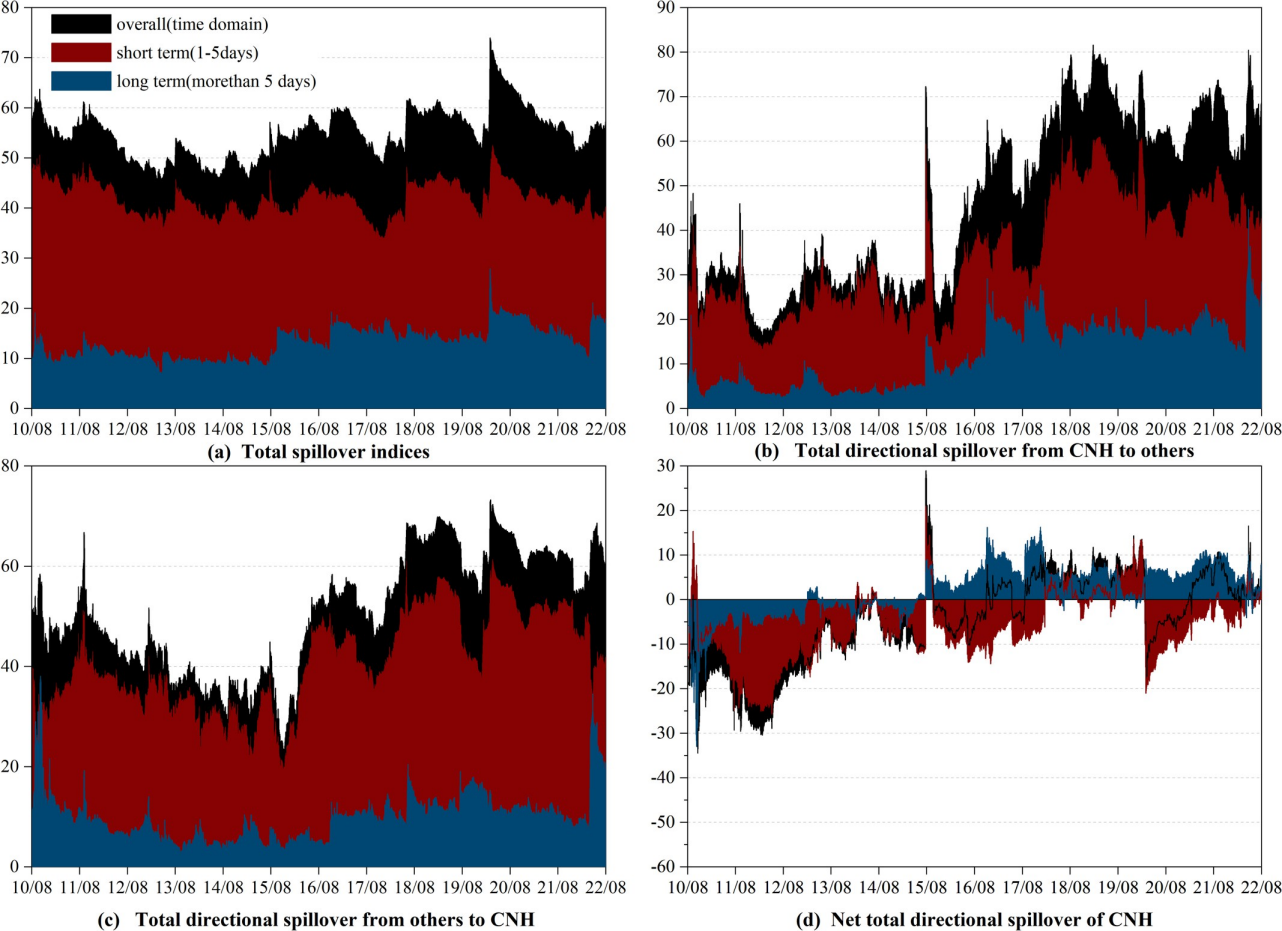
Robust of spillover between CNH and currencies of RCEP members (*p* = 3, *H* = 100). (i) "overall" refers to the spillover observed in the time domain, which is represented by the black shade. (ii) "short term" and "long term" refer to the spillovers observed in the high and low frequency bands.

**Fig 5:**
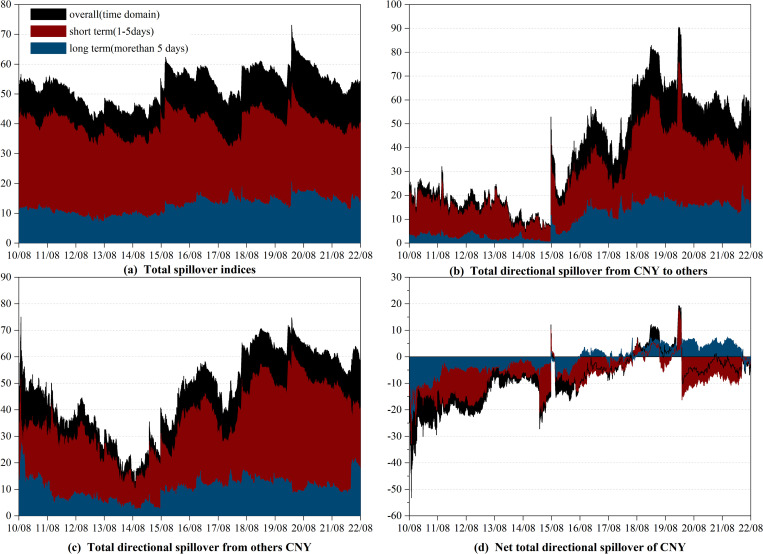
Robust of spillover between CNY and currencies of RCEP members (*p* = 1, *H* = 85). (i) "overall" refers to the spillover observed in the time domain, which is represented by the black shade. (ii) "short term" and "long term" refer to the spillovers observed in the high and low frequency bands.

**Fig 6:**
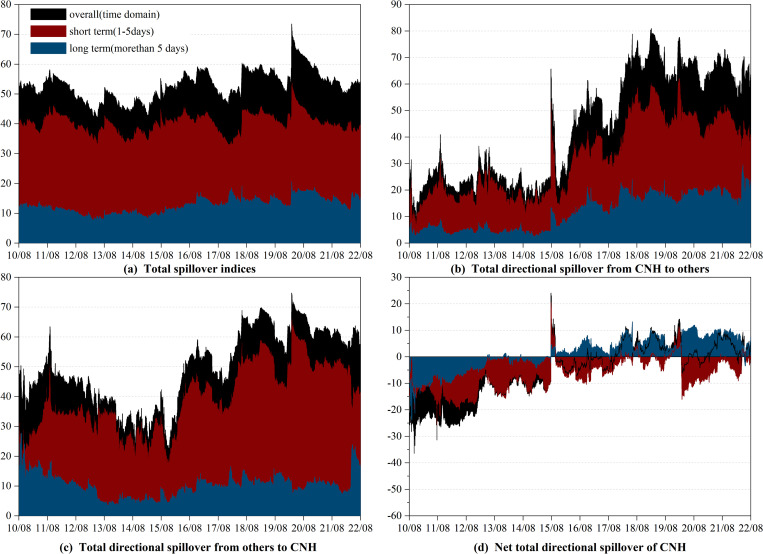
Robust of spillover between CNH and currencies of RCEP members (*p* = 1, *H* = 85). (i) "overall" refers to the spillover observed in the time domain, which is represented by the black shade. (ii) "short term" and "long term" refer to the spillovers observed in the high and low frequency bands.

Secondly, considering that the spillover may be sensitive to the lag length (*p*) and forecasting horizon (*H*) [5, 8], we conduct a robustness test by varying these parameters. In Figs 3 and 4, we change the lag length (*p*) to 3 and keep the forecasting horizon (*H*) at 100, while in Figs 5 and 6, we adjust the forecasting horizon (*H*) to 85 and retain the lag length (*p*) at 1. Compared to the results in section 3.2, there are no significant changes in the dynamic spillover effects. Judging from the dynamic evolution of the total spillover, it is sensitive to international economic and political events and shows an upward trend during the sample period. From the perspective of the dynamic spillovers of RMB, the “8.11” RMB exchange rate regime reform, the China-US trade war, and the COVID-19 pandemic all increased the net total directional spillover of RMB. However, it is evident that the evolution of the net total directional spillover of CNH and CNY is different in detail. Following the “8.11” RMB exchange rate regime reform, CNH has been playing the role of a net transmitter in the long term, while CNY has alternated between a net transmitter and a net receiver. Thus, our findings in Figs 3 to 6 suggest that the evolution of the spillover effect does not change significantly when we use different lag lengths (*p*) and forecasting horizons (*H*). The slight change is only shown by the fact that the spillover effect fluctuates a little more sharply when the lag is longer.

In summary, the results in this section using different empirical methods remain largely consistent with the empirical findings in the main text and can demonstrate the good robustness of the main conclusions of this paper.

## 4. Conclusions and implications

The correlation between economic integration and financial integration in global and regional development has consistently been demonstrated [4]. Therefore, it is inevitable for the currencies of RCEP member countries to interrelate. As China constitutes the largest economy and a major trade contributor in the RCEP region, it is imperative to conduct an in-depth investigation into the spillover effects of the RMB exchange rate among the RCEP member countries. In this paper, we study the spillover between RMB and ten major currencies of RCEP member countries from a time-frequency domain perspective using daily data. We have obtained some valuable findings as follows:

Firstly, the currencies in the RCEP region display a strong interaction, as demonstrated by the total spillover indices, which range from 40% to 75%. The interactions exhibit heightened sensitivity to global economic fundamentals, which results in an increase of spillover effects during significant events. In terms of frequency bands, the total spillover effect is more susceptible to short-term factors, indicating that the market processes information promptly and efficiently.

Secondly, the spillover effect of the RMB shows heterogeneity across various frequency bands and countries. In terms of frequency bands, RMB tends to be a net receiver in the short term, but it primarily functions as a net transmitter in the long term, implying that the influence of RMB is mainly driven by long-term factors, such as international trade and foreign direct investment. Concerning countries, the primary net receivers of information from RMB are JPY and VND, while SGD and AUD primarily transmit net spillover to RMB.

Moreover, both domestic exchange rate regime reforms and external political and economic shocks increase the net spillover effect of the RMB, which may be reflected in short-term connectedness or captured by long-term connectedness. Following the "8.11" RMB exchange rate regime reform, the RMB changed from being a complete net receiver to an alternating status of being a net receiver and a net transmitter. The China-US trade war has heightened the long-term impact of RMB, while the COVID-19 pandemic contributes to short-term influence.

Finally, the net spillover effect of CNH consistently surpasses that of CNY, indicating that CNH wields greater influence than CNY. Nevertheless, in times of significant market turbulence, the directional spillover of CNY to the currencies of RCEP members exhibits a more significant ascent. With the opening of China’s capital account and the development of the onshore RMB market, the influence of CNH may gradually diminish.

The above findings have important implications for both policymakers and market participants. On the one hand, market regulators should pay due attention to risk contagion in the foreign exchange market, especially during the outbreak of international political and economic events, strengthen risk prevention, and further promote cooperation among member countries under the RCEP framework. On the other hand, for investors, it is necessary to focus on preventing the input risks arising from the occurrence of risk events and identify the frequency bands of risk contagion to achieve investment risk management. In addition, our research shows that the RMB mainly assumes the role of a net transmitter in the long-term, while it is mostly a net receiver in the short-term. Therefore, we should continue to deepen the market-oriented reform of the RMB exchange rate and further increase the exchange rate’s flexibility to enhance the net transmission capacity of RMB in the short term.

There are several limitations in this study. Firstly, due to the unavailability of data, the Lao kip and Myanmar Kyat were not included in our analysis. In the future, if more relevant data becomes available, we aim to construct a more comprehensive spillover network. Secondly, this paper solely focused on examining the spillover effect of the RMB exchange rate among RCEP member countries and did not provide an econometric analysis of the factors that may affect the spillover. Future research could investigate these factors, potentially leading to new insights.

## Supporting information

S1 File Raw data. (XLSX)

## References

[1] Zhang W, Cao S, Zhang X, Qu X. COVID-19 and stock market performance: Evidence from the RCEP countries. International Review of Economics & Finance. 2023;83:717–35. 10.1016/j.iref.2022.10.013.

[2] Petri PA, Plummer MG. East Asia decouples from the United States: Trade war, COVID-19, and East Asia’s new trade blocs. 2020. Available from: https://ideas.repec.org/p/iie/wpaper/wp20-09.html.

[3] Jia Z, Wang Y, Chen Y, Chen Y. The role of trade liberalization in promoting regional integration and sustainability: The case of regional comprehensive economic partnership. PLOS ONE. 2022;17(11):e0277977. doi: 10.1371/journal.pone.0277977 36417481 PMC9683547

[4] Zhang B, Zhou P. Financial development and economic growth in a microfounded small open economy model. The North American Journal of Economics and Finance. 2021;58:101544. 10.1016/j.najef.2021.101544.

[5] Wen T, Wang G-J. Volatility connectedness in global foreign exchange markets. Journal of Multinational Financial Management. 2020;54:100617. 10.1016/j.mulfin.2020.100617.

[6] Zhang C, Chen P. Applying the three-stage SBM-DEA model to evaluate energy efficiency and impact factors in RCEP countries. Energy. 2022;241:122917. 10.1016/j.energy.2021.122917.

[7] Subramanian A, Kessler M. The Renminbi Bloc is Here: Asia Down, Rest of the World to Go? Journal of Globalization and Development. 2013;4(1):49–94. 10.1515/jgd-2013-0017.

[8] Wei Z, Luo Y, Huang Z, Guo K. Spillover effects of RMB exchange rate among B&R countries: Before and during COVID-19 event. Finance Research Letters. 2020;37:101782. 10.1016/j.frl.2020.101782. 33013238 PMC7525334

[9] Shu C, He D, Dong J, Wang H. Regional pull vs global push factors: China and US influence on Asian financial markets. Journal of International Money and Finance. 2018;87:112–32. 10.1016/j.jimonfin.2018.04.004.

[10] Kočenda E, Moravcová M. Exchange rate comovements, hedging and volatility spillovers on new EU forex markets. Journal of International Financial Markets, Institutions and Money. 2019;58:42–64. 10.1016/j.intfin.2018.09.009.

[11] Shu C, He D, Cheng X. One currency, two markets: the renminbi’s growing influence in Asia-Pacific. China Economic Review. 2015;33:163–78. 10.1016/j.chieco.2015.01.013.

[12] Bouri E, Demirer R, Gabauer D, Gupta R. Financial market connectedness: The role of investors’ happiness. Finance Research Letters. 2022;44:102075. doi: 10.1016/j.frl.2021.102075

[13] Keddad B, Sato K. The influence of the renminbi and its macroeconomic determinants: A new Chinese monetary order in Asia? Journal of International Financial Markets, Institutions and Money. 2022;79:101586. 10.1016/j.intfin.2022.101586.

[14] Shahzad SJH, Kyei CK, Gupta R, Olson E. Investor sentiment and dollar-pound exchange rate returns: Evidence from over a century of data using a cross-quantilogram approach. Finance Research Letters. 2021;38:101504. 10.1016/j.frl.2020.101504.

[15] Plakandaras V, Papadimitriou T, Gogas P, Diamantaras K. Market sentiment and exchange rate directional forecasting. Algorithmic Finance. 2015;4:69–79. 10.3233/AF-150044.

[16] Singh VK. Day-of-the-week effect of major currency pairs: new evidences from investors’ fear gauge. Journal of Asset Management. 2019;20(7):493–507. 10.1057/s41260-019-00140-6.

[17] Chau F, Deesomsak R, Lau MCK. Investor sentiment and feedback trading: Evidence from the exchange-traded fund markets. International Review of Financial Analysis. 2011;20(5):292–305. 10.1016/j.irfa.2011.06.006.

[18] Baruník J, Křehlík T. Measuring the Frequency Dynamics of Financial Connectedness and Systemic Risk*. Journal of Financial Econometrics. 2018;16(2):271–96. 10.1093/jjfinec/nby001.

[19] Anwer Z, Naeem MA, Hassan MK, Karim S. Asymmetric connectedness across Asia-Pacific currencies: Evidence from time-frequency domain analysis. Finance Research Letters. 2022;47:102782. 10.1016/j.frl.2022.102782.

[20] Fischer C. Determining global currency bloc equilibria: An empirical strategy based on estimates of anchor currency choice. Journal of International Money and Finance. 2016;64:214–38. 10.1016/j.jimonfin.2016.02.019.

[21] Guo D, Zhou P. The rise of a new anchor currency in RCEP? A tale of three currencies. Economic Modelling. 2021;104:105647. 10.1016/j.econmod.2021.105647.

[22] Baruník J, Kočenda E, Vácha L. Asymmetric volatility connectedness on the forex market. Journal of International Money and Finance. 2017;77:39–56. 10.1016/j.jimonfin.2017.06.003.

[23] Shahzad SJH, Arreola-Hernandez J, Rahman ML, Uddin GS, Yahya M. Asymmetric interdependence between currency markets’ volatilities across frequencies and time scales. International Journal of Finance & Economics. 2021;26(2):2436–57. doi: 10.1002/ijfe.1915 WOS:000557790200001

[24] Albulescu CT, Aubin C, Goyeau D, Tiwari AK. Extreme co-movements and dependencies among major international exchange rates: A copula approach. The Quarterly Review of Economics and Finance. 2018;69:56–69. doi: 10.1016/j.qref.2018.03.007

[25] Tamakoshi G, Hamori S. Co-movements among major European exchange rates: A multivariate time-varying asymmetric approach. International Review of Economics & Finance. 2014;31:105–13. 10.1016/j.iref.2014.01.016.

[26] Salisu AA, Oyewole OJ, Fasanya IO. Modelling return and volatility spillovers in global foreign exchange markets. Journal of Information and Optimization Sciences. 2018;39(7):1417–48. 10.1080/02522667.2017.1367507.

[27] Salisu AA, Ayinde TO. Testing for spillovers in naira exchange rates: The role of electioneering & global financial crisis. Borsa Istanbul Review. 2018;18(4):341–8. 10.1016/j.bir.2018.07.007.

[28] Ito T. A new financial order in Asia: Will a RMB bloc emerge? Journal of International Money and Finance. 2017;74:232–57. 10.1016/j.jimonfin.2017.02.019.

[29] Kumar S, Tiwari AK, Chauhan Y, Ji Q. Dependence structure between the BRICS foreign exchange and stock markets using the dependence-switching copula approach. International Review of Financial Analysis. 2019;63:273–84. 10.1016/j.irfa.2018.12.011.

[30] Dua P, Tuteja D. Financial crises and dynamic linkages across international stock and currency markets. Economic Modelling. 2016;59:249–61. https://doi.org/10.1016/j.econmod.2016.07.013.

[31] Antonakakis N, Cunado J, Filis G, Gabauer D, de Gracia FP. Oil and asset classes implied volatilities: Investment strategies and hedging effectiveness. Energy Economics. 2020;91:104762. https://doi.org/10.1016/j.eneco.2020.104762.

[32] Singh VK, Nishant S, Kumar P. Dynamic and directional network connectedness of crude oil and currencies: Evidence from implied volatility. Energy Economics. 2018;76:48–63. https://doi.org/10.1016/j.eneco.2018.09.018.

[33] Boubakri S, Guillaumin C, Silanine A. Non-linear relationship between real commodity price volatility and real effective exchange rate: The case of commodity-exporting countries. Journal of Macroeconomics. 2019;60:212–28. https://doi.org/10.1016/j.jmacro.2019.02.004.

[34] Singhal S, Choudhary S, Biswal PC. Return and volatility linkages among International crude oil price, gold price, exchange rate and stock markets: Evidence from Mexico. Resources Policy. 2019;60:255–61. https://doi.org/10.1016/j.resourpol.2019.01.004.

[35] Al-Shboul M, Assaf A, Mokni K. Does economic policy uncertainty drive the dynamic spillover among traditional currencies and cryptocurrencies? The role of the COVID-19 pandemic. Research in International Business and Finance. 2023;64:101824. doi: 10.1016/j.ribaf.2022.101824 36474632 PMC9715263

[36] Frankel JA, Wei S-J. Yen bloc or dollar bloc? Exchange rate policies of the East Asian economies. In: Ito T, Krueger AO, editors. Macroeconomic linkage: Savings, exchange rates, and capital flows. Chicago: University of Chicago 1994. pp. 295–333.

[37] Marconi D. Currency comovements in Asia-Pacific: The regional role of the renminbi. Pacific Economic Review. 2018;23(2):150–63. 10.1111/1468-0106.12266. WOS:000432025600003

[38] Ito T. Influence of the renminbi on exchange rate policies of other Asian currencies. In: Goldstein M, Lardy NR, editors. Debating China’s exchange rate policy. Washington: Peterson Institute for International Economics; 2008. pp. 239–58.

[39] Liu T, Wang X, Woo WT. The rise of Renminbi in Asia: Evidence from Network Analysis and SWIFT dataset. Journal of Asian Economics. 2022;78 doi: 10.1016/j.asieco.2021.101431 WOS:000788099300003

[40] Eichengreen B, Lombardi D. RMBI or RMBR? Is the Renminbi Destined to Become a Global or Regional Currency? Asian Economic Papers. 2017;16(1):35–59. 10.1162/ASEP_a_00483.

[41] Kawai M, Pontines V. Is there really a renminbi bloc in Asia?: A modified Frankel–Wei approach. Journal of International Money and Finance. 2016;62:72–97. 10.1016/j.jimonfin.2015.12.003.

[42] Kim CS, Kim S, Wang Y. RMB Bloc in East Asia: Too Early to Talk About It? Asian Economic Papers. 2018;17(3):31–48. 10.1162/asep_a_00628.

[43] Thorbecke W. East Asian value chains, exchange rates, and regional exchange rate arrangements. Journal of Asian Economics. 2019;65:101132. 10.1016/j.asieco.2019.101132.

[44] Keddad B. How do the Renminbi and other East Asian currencies co-move? Journal of International Money and Finance. 2019;91:49–70. 10.1016/j.jimonfin.2018.11.003.

[45] Diebold FX, Yilmaz K. Better to give than to receive: Predictive directional measurement of volatility spillovers. International Journal of Forecasting. 2012;28(1):57–66. 10.1016/j.ijforecast.2011.02.006.

[46] Diebold FX, Yılmaz K. On the network topology of variance decompositions: Measuring the connectedness of financial firms. Journal of Econometrics. 2014;182(1):119–34. 10.1016/j.jeconom.2014.04.012.

[47] Zhou Y, Cheng X, Wang Y. Measuring the importance of RMB in the exchange rate spill-over networks: new indices of RMB internationalisation. Economic and Political Studies. 2020;8(3):331–54. 10.1080/20954816.2020.1775374.

[48] Chow HK. Connectedness of Asia Pacific forex markets: China’s growing influence. International Journal of Finance & Economics. 2021;26(3):3807–18. 10.1002/ijfe.1988. WOS:000555530100001

[49] Barunik J, Kocenda E, Vacha L. Volatility Spillovers Across Petroleum Markets. Energy Journal. 2015;36(3):309–29. 10.5547/01956574.36.3.jbar. WOS:000356466100014

[50] Barunik J, Kocenda E, Vacha L. Asymmetric connectedness on the US stock market: Bad and good volatility spillovers. Journal of Financial Markets. 2016;27:55–78. 10.1016/j.finmar.2015.09.003. WOS:000370583800003

[51] Adekoya OB, Akinseye AB, Antonakakis N, Chatziantoniou I, Gabauer D, Oliyide J. Crude oil and Islamic sectoral stocks: Asymmetric TVP-VAR connectedness and investment strategies. Resources Policy. 2022;78:102877. 10.1016/j.resourpol.2022.102877.

[52] Li JY, Liu RR, Yao YZ, Xie QW. Time-frequency volatility spillovers across the international crude oil market and Chinese major energy futures markets: Evidence from COVID-19. Resources Policy. 2022;77 10.1016/j.resourpol.2022.102646. WOS:000819876300012

[53] Su XF, Li Y. Dynamic sentiment spillovers among crude oil, gold, and Bitcoin markets: Evidence from time and frequency domain analyses. Plos One. 2020;15(12) WOS:000597149100136 doi: 10.1371/journal.pone.0242515 33270645 PMC7714240

[54] Jiang C, Wu YF, Li XL, Li X. Time-frequency Connectedness between Coal Market Prices, New Energy Stock Prices and CO2 Emissions Trading Prices in China. Sustainability. 2020;12(7):2823. 10.3390/su12072823.

[55] Chatziantoniou I, Gabauer D, Stenfors A. Interest rate swaps and the transmission mechanism of monetary policy: A quantile connectedness approach. Economics Letters. 2021;204:109891. 10.1016/j.econlet.2021.109891.

[56] Ando T, Greenwood-Nimmo M, Shin Y. Quantile Connectedness: Modeling Tail Behavior in the Topology of Financial Networks. Management Science. 2022;68(4):2401–31. 10.1287/mnsc.2021.3984. WOS:000798270800002

[57] Chatziantoniou I, Gabauer D, Perez de Gracia F. Tail risk connectedness in the refined petroleum market: A first look at the impact of the COVID-19 pandemic. Energy Economics. 2022;111:106051. 10.1016/j.eneco.2022.106051.

[58] Cunado J, Chatziantoniou I, Gabauer D, de Gracia FP, Hardik M. Dynamic spillovers across precious metals and oil realized volatilities: Evidence from quantile extended joint connectedness measures. Journal of Commodity Markets. 2023;30:100327. 10.1016/j.jcomm.2023.100327.

[59] Lastrapes WD, Wiesen TFP. The joint spillover index. Economic Modelling. 2021;94:681–91. 10.1016/j.econmod.2020.02.010.

[60] Balcilar M, Gabauer D, Umar Z. Crude Oil futures contracts and commodity markets: New evidence from a TVP-VAR extended joint connectedness approach. Resources Policy. 2021;73:102219. 10.1016/j.resourpol.2021.102219.

[61] Antonakakis N, Chatziantoniou I, Gabauer D. Refined Measures of Dynamic Connectedness based on Time-Varying Parameter Vector Autoregressions. Journal of Risk and Financial Management 2020;13(4):84. 10.3390/jrfm13040084.

[62] Chatziantoniou I, Gabauer D, Gupta R. Integration and Risk Transmission in the Market for Crude Oil: A Time-Varying Parameter Frequency Connectedness Approach. 2021; WP21:47. Available from: https://www.up.ac.za/media/shared/61/WP/wp_2021_47.zp209709.pdf.

[63] Gruić B, Mojon B, Packer F, Schrimpf A, Shin HS, Sushko VJBQR. The internationalisation of EME currency trading. 2022:49–61. Available from: https://www.bis.org/publ/qtrpdf/r_qt2212g.pdf.

[64] Koop G, Korobilis D. A new index of financial conditions. European Economic Review. 2014;71:101–16. 10.1016/j.euroecorev.2014.07.002.

[65] Tiwari AK, Cunado J, Gupta R, Wohar ME. Volatility spillovers across global asset classes: Evidence from time and frequency domains. The Quarterly Review of Economics and Finance. 2018;70:194–202. 10.1016/j.qref.2018.05.001.

[66] Bouri E, Lucey B, Saeed T, Vo XV. Extreme spillovers across Asian-Pacific currencies: A quantile-based analysis. International Review of Financial Analysis. 2020;72:101605. 10.1016/j.irfa.2020.101605.

[67] Zhao YP, Umar Z, Vo XV. Return and volatility connectedness of Chinese onshore, offshore, and forward exchange rate. Journal of Futures Markets. 2021;41(11):1843–60. 10.1002/fut.22243. WOS:000674436000001

[68] Qin J. Relationship between onshore and offshore renminbi exchange markets: Evidence from multiscale cross-correlation and nonlinear causal effect analyses. Physica a-Statistical Mechanics and Its Applications. 2019;527 10.1016/j.physa.2019.121183. WOS:000480625700115

[69] Ruan Q, Bao J, Zhang M, Fan L. The effects of exchange rate regime reform on RMB markets: A new perspective based on MF-DCCA. Physica A: Statistical Mechanics and its Applications. 2019;522:122–34. 10.1016/j.physa.2019.01.110.

